# Harmonizing evidence-based practice, implementation context, and implementation strategies with user-centered design: a case example in young adult cancer care

**DOI:** 10.1186/s43058-021-00147-4

**Published:** 2021-04-26

**Authors:** Emily R. Haines, Alex Dopp, Aaron R. Lyon, Holly O. Witteman, Miriam Bender, Gratianne Vaisson, Danielle Hitch, Sarah Birken

**Affiliations:** 1 Department of Social Sciences and Health Policy, Wake Forest School of Medicine, 525 Vine Street, Winston-Salem, NC 27101 USA; 2 Department of Behavioral and Policy Sciences, RAND Corporation, 1776 Main St, Santa Monica, CA 90401 USA; 3 Psychiatry and Behavioral Sciences, University of Washington, 6200 NE 74th Street, Suite 100, Seattle, WA 98115 USA; 4 Department of Family and Emergency Medicine, Faculty of Medicine, Laval University, Ferdinand Vandry Pavillon, 1050 Avenue de la Médecine,, Quebec City, QC G1V 0A6 Canada; 5 Sue & Bill Gross School of Nursing, University of California, Irvine, 252C Berk Hall, Irvine, CA 92697-3959 USA; 6 Occupational Therapy, Faculty of Medicine, Laval University, Ferdinand Vandry Pavillon, 1050 Avenue de la Médecine, Quebec City, QC G1V 0A6 Canada; 7 Department of Physical Activity and Nutrition Research, School of Health and Social Development, Deakin University, Waterfront Campus, 1 Gheringhap Street, Geelong, VIC 3220 Australia; 8 Department of Implementation Science, Wake Forest School of Medicine, 525@Vine Room 5219, Medical Center Boulevard, Winston-Salem, NC 27157 USA

**Keywords:** User-centered design, Human-centered design, Context, Evidence-based practice implementation, Designing implementation strategies, Contextual appropriateness, EBP redesign, Stakeholder engagement, Adaptation

## Abstract

**Background:**

Attempting to implement evidence-based practices in contexts for which they are not well suited may compromise their fidelity and effectiveness or burden users (e.g., patients, providers, healthcare organizations) with elaborate strategies intended to force implementation. To improve the fit between evidence-based practices and contexts, implementation science experts have called for methods for adapting evidence-based practices and contexts and tailoring implementation strategies; yet, methods for considering the dynamic interplay among evidence-based practices, contexts, and implementation strategies remain lacking. We argue that harmonizing the three can be facilitated by user-centered design, an iterative and highly stakeholder-engaged set of principles and methods.

**Methods:**

This paper presents a case example in which we used a three-phase user-centered design process to design and plan to implement a care coordination intervention for young adults with cancer. Specifically, we used *usability testing* to redesign and augment an existing patient-reported outcome measure that served as the basis for our intervention to optimize its usability and usefulness, *ethnographic contextual inquiry* to prepare the context (i.e., a comprehensive cancer center) to promote receptivity to implementation, and iterative *prototyping workshops with a multidisciplinary design team* to design the care coordination intervention and anticipate implementation strategies needed to enhance contextual fit.

**Results:**

Our user-centered design process resulted in the Young Adult Needs Assessment and Service Bridge (NA-SB), including a patient-reported outcome measure and a collection of referral pathways that are triggered by the needs young adults report, as well as implementation guidance. By ensuring NA-SB directly responded to features of users and context, we designed NA-SB *for implementation*, potentially minimizing the strategies needed to address misalignment that may have otherwise existed. Furthermore, we designed NA-SB *for scale-up*; by engaging users from other cancer programs across the country to identify points of contextual variation which would require flexibility in delivery, we created a tool intended to accommodate diverse contexts.

**Conclusions:**

User-centered design can help maximize usability and usefulness when designing evidence-based practices, preparing contexts, and informing implementation strategies—in effect, harmonizing evidence-based practices, contexts, and implementation strategies to promote implementation and effectiveness.

**Supplementary Information:**

The online version contains supplementary material available at 10.1186/s43058-021-00147-4.

Contributions to the literature

- Novel approaches are needed to harmonize *evidence-based practices*, the *contexts* in which they are implemented, and the *implementation strategies* intended to facilitate their implementation, thus minimizing the burden on patients, providers, and healthcare organizations while optimizing implementation.
- User-centered design can be leveraged by implementation scientists to (a) optimize *EBP* design to improve key determinants of implementation like usability and usefulness, (b) prepare *context* to promote receptivity toward EBPs (e.g., modifying workflows to accommodate EBP), and (c) select or design *implementation strategies* which increase the contextual appropriateness of an EBP.

## Background

Evidence-based practice (EBP) implementation is often challenged by the poor fit between EBPs and their implementation contexts (i.e., the “set[s] of characteristics and circumstances that consist of active and unique factors, within which the implementation is embedded”) [1, 2]. the use of an *EBP* (i.e., practice with proven efficacy and effectiveness, including interventions, policies, assessments [3]) in a context for which it is not well-suited can compromise its effectiveness and burden users (e.g., patients, providers, healthcare organizations) with elaborate strategies intended to force implementation. However, EBPs are seldom designed to address the nuances of multiple, varying, complex, and changing practice contexts [1]. To accommodate nuanced contexts, EBP developers may produce increasingly complex EBPs [4], resulting in EBPs “that are ultimately too expensive, impractical, or even impossible to construct within real-world constraints” [5].

Despite consistent recognition that there is no implementation without some adaptation, methods to inform systematic EBP adaptation are in their infancy [6, 7]. Implementation scientists have identified various EBP characteristics that influence implementation [8]; such evidence may inform efforts to adapt EBPs to improve implementation. However, the relationship between EBP characteristics and implementation outcomes varies across EBPs and contexts [8], and the same EBP may demonstrate varying degrees of effectiveness in achieving the desired patient outcomes across different contexts [9]. All of this suggests that an EBP’s implementation and effectiveness are inextricably linked to the dynamic and multilevel *contexts* in which they are implemented [10]. Methods for considering the dynamic interplay between EBP and context have not been well articulated [6, 11].

To address discordance between EBPs and contexts, implementation scientists often turn to *implementation strategies*—i.e., “methods or techniques used to enhance the adoption, implementation, and sustainability” of EBPs [12, 13]. However, a “more is better” approach to deploying implementation strategies to compensate for poor EBP-context fit may burden EBP users. Moreover, implementation strategies have often shown only modest effect sizes [14]. For example, in a synthesis of systematic review findings on the effectiveness of clinical guideline implementation strategies, the authors concluded that the evidence base was modest [15]. Similarly, a systematic review of audit and feedback interventions found only small effect sizes [16]. These findings may be in part due to an insufficient consideration of key determinants, such as contextual appropriateness, when selecting or designing implementation strategies [17]. To this end, implementation scientists have called for methods for tailoring implementation strategies to EBPs and contexts [17, 18].

**Fig. 1:**
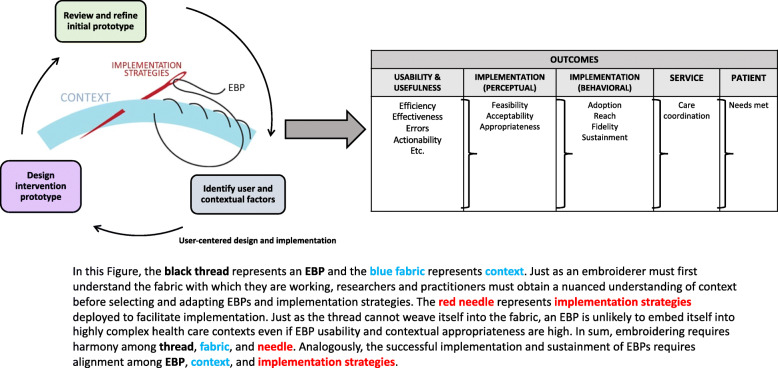
Conceptual model of user-centered design and implementation

Rather than deploying cumbersome EBPs or implementation strategies to improve EBP-context fit, implementation scientists should seek to harmonize EBPs, contexts, and strategies (i.e., design each with respect to the other two). An analogy (Fig. 1) helps illustrate this harmonization: in embroidery, decisions about fabric, needle, or thread are interdependent. For example, a lightweight fabric and thin thread demand a smaller needle, using a large needle may damage the lightweight fabric and thin thread. Likewise, a too-thin thread may break if used with a thick needle or heavy fabric. Depending on the thread count, the fabric may require a stabilizer or alteration before embroidering. Similarly, an *EBP* (i.e., the thread), *context* (i.e., the fabric), and *implementation strategies* (i.e., the needle) should be harmonized to minimize user burden and optimize implementation. “Threading the needle” requires designing EBPs and implementation strategies that are aligned with key features of context.

There is a critical need for the development of “relational and dynamic approaches to theorizing the complex interplay between the characteristics of interventions, the activities of implementers, and the properties of variable broader contexts” [19]. Indeed, advancing methods for harmonizing EBPs, contexts, and implementation strategies have been articulated as a priority for implementation research [8, 20]. Here, we argue that such harmonizing may be facilitated with user-centered design (UCD), an iterative and highly stakeholder-engaged process for designing EBPs, preparing contexts, and informing implementation strategies. To demonstrate, we present a case example in young adult cancer care. Specifically, we describe a three-phase UCD process—(1) *usability testing* (optimizing the thread—i.e., EBP), (2) *ethnographic contextual inquiry* (understanding and preparing the fabric—i.e., context), and (3) *prototyping with a multidisciplinary design team* (threading the needle—i.e., designing EBP and implementation strategies)—to design a care coordination intervention for implementation in a comprehensive cancer center.

### User-centered design

UCD, which is closely related to and often used interchangeably with the term “human-centered design” [21], is an iterative and highly stakeholder-engaged process for creating products which are directly responsive to their intended users and users’ contexts [22]. The primary goals of UCD are improving EBP *usability* (the ease with which it can be successfully used [23]) and *usefulness* (the extent to which it does what it is intended to do [24]). Usability and usefulness are theorized proximal determinants of perceptual implementation outcomes (i.e., acceptability, feasibility, and appropriateness; e.g., usability promotes acceptability) through which they also influence distal behavioral implementation outcomes (e.g., acceptability promotes reach) [25].

Most UCD definitions and frameworks share a common set of principles that contribute to harmonizing EBPs, contexts, and implementation strategies: (1) engaging prospective users to achieve a nuanced understanding of *context*, (2) refining *EBPs* based on user input to optimize usability and usefulness [26], and (3) a multidisciplinary design team collaborating to produce *design and implementation prototypes*. Together, these steps comprise an iterative cycle in which an EBP’s design and implementation strategies are refined until optimized for a given context [27]. For each of these steps, UCD offers myriad methods [26] and strategies [28] for harmonizing EBPs, contexts, and implementation strategies (summarized in Table 1). Although some of UCD’s discrete methods and principles resemble those traditionally used in implementation science (e.g., stakeholder engagement), UCD is unique in its offering of an extensive suite of methods that may be leveraged to refine EBPs, contexts, and implementation strategies. We present UCD as one promising set of approaches implementation scientists may consider drawing upon to promote adoption, implementation, and sustainment.

**Table 1:** Potential applications of UCD in implementation science

Construct	Definition	What UCD offers
**Evidence-based practice** (*the thread*)	Interventions with demonstrated efficacy and effectiveness including programs, actions, processes, policies, and guidelines [3]	• Selecting EBPs that are appropriate for users and their context (e.g., by leveraging UCD measures of usability such as *the System Usability Scale* [29]) • Redesigning EBPs to better fit users and their context (e.g., conducting *usability test* or *heuristic evaluation* to identify an EBP’s design limitations)
**Context** (*the fabric*)	Set of characteristics and circumstances that consist of active and unique factors, within which the implementation is embedded including the following: • Inner (i.e., intra-organizational) context [30] • Outer (i.e., extra-organizational) context [30]	• Assessing context (e.g., conducting *ethnography* or developing *user experience models*) • Preparing context to promote receptivity to EBP (e.g., using *workflow mapping* to modify the workflow to accommodate EBP implementation)
**Implementation strategies** (*the needle*)	Methods or techniques used to enhance the adoption, implementation, and sustainability of an EBP [12]	• Anticipating needed implementation strategies based on context assessment (e.g., conducting *design workshops* to identify areas where fit between EBP and context is low and problem-solving accordingly) • Selecting strategies that are appropriate given EBP and context (e.g., using the *Cognitive Walkthrough for Implementation Strategies* [31] to assess strategy usability) • Tailoring/designing strategies for EBP and context (e.g., by conducting iterative *co-creation sessions* with users)

Figure 1 illustrates the potential of UCD for EBP-context implementation strategy harmonization (i.e., design of each with respect to the other two). In this conceptual model, this harmonization promotes an EBPs’ usability and usefulness and, subsequently, implementation (e.g., acceptability and, in turn, reach), thus limiting demand for implementation strategies. When combined with Proctor’s framework [32], this framework suggests UCD’s potential to improve an EBPs’ service and patient outcomes*.*

## Methods

### Case example: implementation of a care coordination intervention for young adults with cancer

#### Background and project objectives

Each year, more than 20,000 young adults between the ages of 18 and 30 are diagnosed with cancer [33]; many of them do not receive services to meet the range of needs they experience during and after cancer treatment [34–38]. Young adults’ unmet needs result in negative outcomes, including higher distress [35, 36], poorer health-related quality of life [39], and higher physical symptom burden [34]. Despite the complexity and scope of their needs, young adults often do not use potentially beneficial services/resources, even when access is not an issue [40–42]. This disconnect between young adult needs and their use of existing services/resources suggests the need for a care coordination model that (1) effectively assesses young adults’ multifaceted, age-specific, individual, and dynamic needs and (2) uses that information to efficiently connect them to services/resources.

**Fig. 2:**
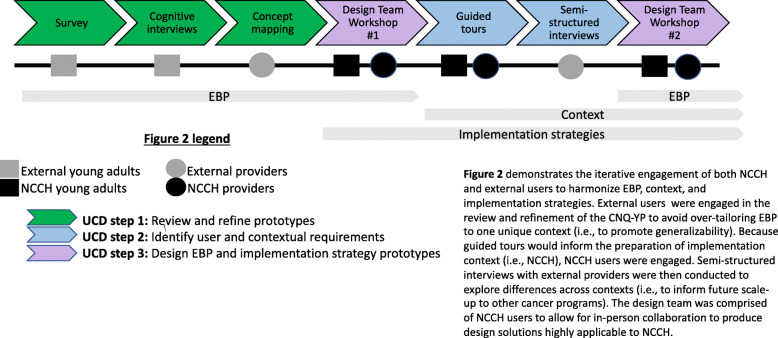
Data collection timeline and users engaged

A substantial step toward this care coordination model was the development of the first multidimensional measure of unmet needs designed specifically for adolescents and young adults: the Cancer Needs Questionnaire - Young People (CNQ-YP) [43, 44]. However, limitations to the usability and usefulness of patient-reported outcome measures like the CNQ-YP (e.g., length, wording ambiguity, redundancy or missing content, lack of connection between identified needs and follow-up actions) have frustrated their real-world implementation and effect on patient outcomes [45, 46]. Despite its potential limitations, we selected the CNQ-YP as a starting point for our intervention because of its specificity to the unique needs of young adults with cancer and because of preliminary evidence pointing to its face and content validity [43]. In this project, we used UCD to redesign the CNQ-YP to optimize its usability and usefulness in the North Carolina Cancer Hospital (NCCH), identify context modifications needed to promote receptivity to its implementation, and anticipate minimally necessary implementation strategies. Our UCD process (Table 2, Fig. 2) produced the Needs Assessment and Service Bridge (NA-SB), a care coordination intervention for young adults with cancer, and a plan for its implementation at NCCH. All procedures were approved by the University of North Carolina’s Institutional Review Board.

**Table 2:** Data collection summary

UCD aim	Method	Deliverable
Review and refine intervention prototype (*the thread*)	Usability testing: • Young adult survey • Cognitive interviews with young adults • Concept mapping with providers/staff	Evidence of the usability and usefulness of the CNQ-YP
Identify user and contextual requirements (*the fabric*)	Ethnographic contextual inquiry: • Guided tours with young adults and providers/staff from NCCH • Semi-structured interviews with providers/staff from external organizations	User and contextual requirements for NA-SB’s delivery and implementation
Design intervention and implementation strategy prototypes based on user and contextual requirements (*threading the needle*)	Design team workshops: • Workshop #1 • Workshop #2	NA-SB prototype and anticipated implementation strategies needed
*Result*		NA-SB + implementation guidance

#### Multidisciplinary design team

In implementation research, stakeholder engagement has sometimes been limited or superficial [47–49]. In contrast, UCD demands an active and iterative approach to engagement, often with the same group of users reviewing prototypes at multiple time points [50]. Thus, at the beginning of the project, we convened an NA-SB *design team* comprised of key stakeholder groups. Throughout the project, the investigator team presented prototypes and other information to the design team and, based on their interactions with prototypes and collaborative discussion, made iterative improvements to NA-SB design and implementation strategies.

Design team members included researchers in cancer care delivery, patient-reported outcomes, UCD, and implementation science (*n*=4) and prospective NA-SB users, including NCCH clinical partners (oncologist; social worker/director of NCCH’s young adult program [*n*=2]) and young adult representatives (*n*=5) nominated by clinical partners. Nominees were primarily individuals who had previously expressed interest in research or advocacy activities related to young adult cancer and, thus, would be more likely to consider the extensive and ongoing participation that joining the design team would entail.

To recruit young adult representatives for the design team, clinical partners connected young adults via email to the project lead (EH). EH provided them with materials including a project summary, a breakdown of their expected role and time commitment, and a brief summary of UCD. EH then met with each young adult interested in participating to discuss the project and develop rapport, then met with them all together to build group rapport. Young adult representatives received a one-time $150 incentive for participation.

### Review and refine prototypes (optimize the thread)

#### Overview

*Usability testing* involves hands-on evaluation of the extent to which a product or innovation can be used by specified users to achieve specified goals; usability testing can be used to iteratively refine EBPS to better align with context [51]. We conducted three rounds of usability testing to examine user interactions with the CNQ-YP: (1) an online survey assessing young adults’ needs and preferences for a needs assessment using the CNQ-YP as a prototype for them to react to, (2) cognitive interviews [52] with young adults to triangulate survey data with in-depth evidence of their perceptions of the CNQ-YP’s usability and usefulness, and (3) concept mapping [53] exercises focused on usefulness, in which young adult providers mapped needs onto services/resources to address the needs.

#### Young adult survey

##### Objectives

The objectives are to identify missing content, streamline redundant or low-priority content, and identify other usability and usefulness concerns.

##### Instrument

The survey instrument (Additional File 1) included three sections: (1) study information, consent, and demographic items (i.e., age, gender, clinical characteristics, social support, educational/vocational status, health insurance status); (2) the CNQ-YP in its original form; and (3) items assessing respondents’ perception of the CNQ-YP. To assess general attitudes toward the tool, we used items from three Likert-type measures of feasibility, acceptability, and appropriateness [54]. We assessed usefulness through two Likert-type items asking (1) the extent to which respondents thought the CNQ-YP accurately captured their needs and (2) the likelihood that they would use services or resources offered to them based on indicated needs. For each of these measures, we qualitatively probed respondents on usability and usefulness issues driving their concerns with the tool’s feasibility, acceptability, or appropriateness.

##### Sample and recruitment

To be included in the survey, we required participants (*n*=100) to be age 18–30 and have been diagnosed with cancer prior to survey administration. Although usability testing can be done with small samples (e.g., *n*=20) [51, 55], our target sample size was *n*=100 because we wanted to achieve breadth in usability data prior to achieving more depth through cognitive interviews. To promote young adult participant diversity (race, ethnicity, age, geographic region, setting of care, etc.), we recruited through key contacts (i.e., leaders of young adult programs and advocacy groups in the USA identified by our clinical partners), social media (i.e., a series of Twitter messages shared by tagging relevant groups and hashtags), and our design team.

##### Procedure

We administered the survey through a secure online platform, Qualtrics (Provo, UT). On average, the survey took 15 min to complete.

##### Analysis

We used descriptive statistics for respondents’ demographics, needs reported on the CNQ-YP tool, and perceptions of the CNQ-YP. To identify emergent themes regarding the CNQ-YP’s usability and usefulness in free-text responses, we used template analysis [56].

#### Cognitive interviews

##### Objective

The objective is to triangulate survey data on CNQ-YP usability and usefulness through a nuanced understanding of content, wording, or comprehension concerns.

##### Interview guide

With input from the design team, we developed the cognitive interview guide to encourage participants to “think aloud” as they read and reflected on the CNQ-YP itemset and probe them to comment on topics such as item content and wording, response options, format, length, comprehensiveness, and repetitiveness (Additional File 2).

##### Sample and recruitment

We purposively sampled from among survey participants. Consistent with cognitive interview methodology [52], the target sample size was small (i.e., *n*=5–10); however, we prioritized demographic variation when sampling to promote NA-SB’s relevance to diverse young adults. We recruited young adults (*n*=5) until we reached thematic saturation, i.e., when subsequent interviews did not generate new information regarding CNQ-YP’s usability or usefulness.

##### Procedure

EH conducted 1-h cognitive interviews (*n*=5) via Zoom, a video-conferencing platform. Interviews were audio-recorded. EH navigated the CNQ-YP through the screen-share function, soliciting participants’ input on each item. At the end of each interview, EH summarized her takeaways with interviewees for the purposes of member checking [57].

##### Analysis

We inductively identified themes, noting concerns related to the CNQ-YP’s usability and usefulness. We then created a table organizing participants’ concerns within each of the identified themes for presentation to the design team during our first workshop (described later).

#### Concept mapping

##### Objective

The objective is to promote the usefulness of the CNQ-YP by grouping needs by services/resources expected to address those needs.

##### Instrument

The design team approved changes to CNQ-YP content based on survey and cognitive interview results. We pre-loaded the resulting list of young adult needs into an online secure platform called Concept Systems Global Max © (CSGM). CSGM included two concept mapping exercises: (1) sorting an electronic deck of cards, each containing a young adult need, into like categories (i.e., “follow-up domains”) that could be addressed by the same service/resource (e.g., needs related to depression and anxiety might be grouped together as potentially addressable by referral to a mental health professional) and (2) rating needs on Likert-type response scales in terms of two key pragmatic properties: *importance* (i.e., severity of consequences if that need goes unmet) and *actionability* (i.e., likelihood that need can be met through a service or resource) [58].

##### Sample and recruitment

Concept mapping participants included cancer program providers (e.g., oncologists, nurses, and social workers) and staff (e.g., program managers and administrators)—i.e., the prospective NA-SB user groups expected to have the most knowledge about service and resource delivery for this population. Recruitment through the key contacts established during survey recruitment was intended to achieve the minimum sample size of *n*=15 needed for concept mapping analyses [59].

##### Procedure

Participants accessed the web-based concept mapping exercises through emailed links to the project in CSGM. The exercises took approximately 30 min to complete.

##### Analysis

CSGM used hierarchical cluster analysis to characterize how participants grouped needs, creating several potential cluster maps based on proximity among needs, where proximal needs were more frequently grouped together as triggering the same follow-up action than distal ones, and “go-zone graphs,” in which needs are displayed as points on a quadrant in terms of their relative importance and actionability. Concept mapping data was presented to the design team for interpretation during the first prototyping workshop (described later).

### Identify user and contextual requirements (understand and prepare the fabric)

#### Overview

We used *contextual inquiry* [60], including ethnographic guided tours [61] and interviews, to gather detailed information about context to inform context modifications needed to promote receptivity to NA-SB implementation and the identification of minimally necessary implementation strategies. In contextual inquiry, which comes from UCD, in-depth data on a few carefully selected individuals provides a fuller picture of users and their context [62]. By documenting naturally occurring user tasks and interactions among patients and providers through in-depth observation, *ethnography*, a promising yet underused method for implementation research [63], provides rich data on implementation context [64, 65], making it useful for contextual inquiry. Ethnographic methods are relevant to UCD because they offer a more nuanced understanding of users and context than traditional questionnaires or interviews, including novel insights on user tasks, attitudes, and interactions with their environment [22, 26, 66]. Additional File 4 includes the Standards for Reporting Qualitative Research (SRQR) checklist adhered to for these data collection activities.

#### Guided tours

##### Objective

The objective is to capture the contextual elements beyond just those which users can verbalize, including details and motivations that have become habitual or implicit to the tasks they perform [67].

##### Instrument

To promote the flexibility required for guided tours [61, 68], we identified potential questions based on four domains of Maguire et al.’s typology of user and contextual factors to consider in UCD from which we could choose: (1) user characteristics, (2) user tasks, (3) physical and technical environment, and (4) organizational environment [26] (Additional File 3).

##### Sample and recruitment

To capture the perspective of potential NA-SB implementers, we conducted guided tours with our clinical partners at NCCH (*n*=2). To capture the patient perspective, we conducted guided tours with young adults ages 18–30 receiving inpatient or outpatient care at NCCH (*n*=10). Consistent with the preferred approach for determining sample size in qualitative research [69], young adults were recruited until thematic saturation was reached, i.e., when subsequent guided tours did not generate new information regarding contextual factors. Our clinical partners at NCCH facilitated the recruitment of young adults for guided tours by distributing a recruitment flyer and connecting EH via email to those interested.

##### Procedure

EH conducted 4-h guided tours with clinical partners as they completed clinical, administrative, and other duties, asking questions about their tasks and thoughts. EH followed young adults and accompanying family members from the moment they entered the hospital for their outpatient appointments until the moment they exited, asking them questions as they interacted with their environment and healthcare professionals, while attempting to minimize participant disruptions. For inpatient guided tours, EH spent 2 h with young adults receiving inpatient care. EH took extensive field notes and audio-recorded portions of the guided tours for which only consenting parties were present. We offered young adult participants a $50 participation incentive.

##### Analysis

We used template analysis, identifying a priori themes based on Maguire’s constructs and allowing for the identification of additional themes [56]. To calibrate our coding schema, EH and a colleague independently coded excerpts from one set of guided tour field notes and interview transcriptions per Maguire constructs; EH proceeded to code the remaining data. For each Maguire domain, we collaboratively synthesized user and contextual factors and created a “translation table” [70], which translated factors into their implications for NA-SB design and implementation (i.e., *user and contextual requirements*). For example, providers reported the importance of integrating new tools into the electronic medical record; we translated this into the requirement that NA-SB interfaces with NCCH’s electronic medical record. All requirements were vetted and prioritized by the design team during the second workshop (see description below).

#### Semi-structured interviews

##### Objectives

The objectives are to review the findings from guided tours with external users and identify any areas of divergence or additional needs or contextual features, thus promoting the generalizability of findings.

##### Interview guide

With input from the design team, we developed a semi-structured interview guide based on Maguire’s typology [26] and guided tour findings.

##### Sample

We conducted semi-structured interviews with young adult providers and advocates who had previously facilitated survey and concept mapping recruitment: program managers (*n*=2) and nurse navigators (*n*=2) serving primarily young adults, and consultants (*n*=2) involved in young adult program development. Given the variation across interviewees’ contexts (e.g., variation by location, institution type, model of young adult care, funding source), we considered the small sample size sufficient to achieve the objective of the interviews, which was to identify potential areas where NA-SB delivery or implementation may differ across contexts.

##### Procedure

EH conducted 1-h semi-structured telephone interviews. At the end of each interview, EH summarized major takeaways for member checking [57]. We audio-recorded and transcribed the interviews verbatim.

##### Analysis

We analyzed the interview data using template analysis [56].

### Design prototypes based on user and contextual requirements (thread the needle)

#### Overview

UCD often involves engaging the design team in prototyping workshops or sessions during which limited versions of the intervention/product are generated collaboratively. This iterative *prototyping* process—which relies on visual cues to digest user data with multiple user groups—represents a novel method for coproduction in implementation science. Through two 3-h workshops, our design team collaboratively redesigned the CNQ-YP (i.e., the thread) with usability and usefulness in mind, redesigned NCCH care processes (i.e., the fabric) to facilitate the tool’s implementation and usefulness in routine care, and anticipated minimally necessary implementation strategies (i.e., the needle). It resulted in NA-SB and a compilation of implementation strategies, each informed by context and designed to account for the other’s characteristics.

#### Design team workshop #1

##### Objective

During the first design team workshop, we used usability testing data to inform the elimination, addition, or refinement of CNQ-YP items. We also used concept mapping data to group needs into follow-up domains.

##### Sample

The sample is design team members.

##### Materials

**Fig. 3:**
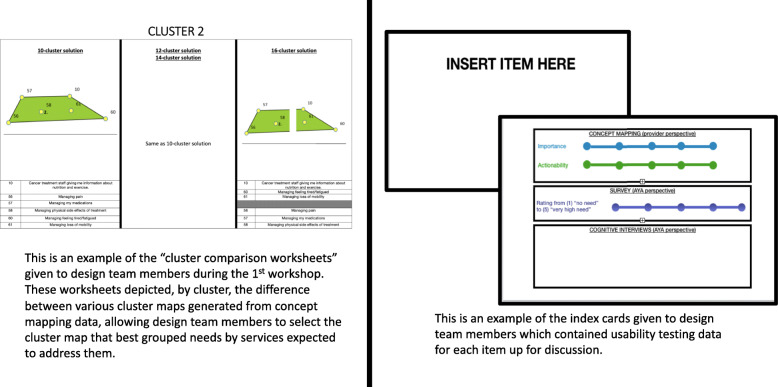
Design team workshop #1 materials (cluster comparison worksheet and usability index cards)

Design team members were given a summary of project information and usability testing results. Additionally, the study team developed index cards representing each item up for discussion (i.e., those for which usability or usefulness issues had been identified), which included usability testing data with respect to that item. We also developed index cards representing potential additional items elicited from usability testing data (see Fig. 3 for an example index card). Finally, attendees were given “cluster comparison worksheets” (see example in Fig. 3) which visually depicted, by cluster, the differences between various cluster maps generated from concept mapping data.

##### Procedure

To begin the design team workshop, EH gave a brief overview of the project and usability testing results. Next, we used index cards and stickers to discuss each item and vote on decisions as to whether that item should be eliminated, added, or revised. Votes were then tallied to arrive at a decision about that particular item; where voting was split (i.e., greater than two design team members in opposition), we discussed further until the design team reached consensus.

Once item revisions were made, we turned our focus toward grouping items into appropriate follow-up domains. We used the “cluster comparison worksheets” to review the concept mapping cluster maps and, through collaborative discussion, selected the most interpretable cluster map. We then moved items between clusters, as needed, and labeled each cluster according to the service that needs in that cluster should trigger. After grouping high-priority needs by follow-up domains, the design team identified services/resources at NCCH which corresponded to each follow-up domain, establishing explicit referral pathways for each domain. We also anticipated implementation strategies needed to facilitate this kind of multidisciplinary service provision through collaborative discussion.

##### Analysis

Detailed notes were taken during design team workshop #1 to capture all discussion points leading to decisions on itemset content; the meeting was also recorded for further elaboration on meeting notes. After the workshop, EH drafted the revised patient-reported outcome measure, grouping needs by follow-up domain, and obtained additional design team feedback via email.

#### Design team workshop #2

##### Overview

After soliciting user and contextual data through guided tours and interviews, we convened the design team for a second workshop during which we presented them the ethnography findings in juxtaposition with the patient-reported outcome measure and referral pathways produced during the first design team workshop. This juxtaposition allowed design team members to anticipate context modifications and needed implementation strategies *with respect to* the redesigned tool itself. Through popular UCD methods, “storyboarding” (i.e., “sequences of images which show the relationship between user actions or inputs and system” [26]), “personas” (i.e., using caricatures of key user groups to convey users’ needs to the design team), and “scenarios of use” (i.e., using specific examples of how users, context, and NA-SB might interact) [26], we collaboratively specified who will deliver the needs assessment, when, how often, and the materials and procedure that will be used to do so. This workshop was also used to plan for NA-SB implementation.

##### Sample

Design team members plus various additional providers involved in young adult care at NCCH (*n*=6) including (1) a pediatric oncology nurse practitioner, (2) a pediatric palliative care social worker, (3) a nurse navigator, (4) a pediatric palliative care physician, (5) a chaplain, and (6) a second young adult social worker. The purpose of including these individuals was to capture the perspectives of the range of provider groups that might interface with NA-SB in practice and also to build buy-in for future NA-SB implementation at NCCH, potentially strengthening referral pathways.

##### Materials

We gave design team participants a packet of information including a project overview, the revised patient-reported outcome measure and referral pathways developed through design team workshop #1, and a summary of ethnography results. Other materials included a storyboard depicting the steps of NA-SB delivery, personas, and scenarios of use, all of which were generated based on ethnography findings. Four personas were crafted to represent four user types: (1) a young adult receiving care in pediatric oncology, (2) a young adult with frequent inpatient stays, (3) a young adult receiving maintenance treatment with appointments occurring less frequently, and (4) a young adult with a prognosis of less than 1 year. Scenarios of use reflected various appointment types and were presented using a flowchart of patients’ appointments. For example, one scenario included labs, treatment, and a clinical appointment; another included just treatment; a third included just a clinical appointment.

##### Procedure

First, we gave an overview of the project and ethnography results. Second, we discussed the ethnography translation table, giving the design team the opportunity to vet the research team’s translation of user and contextual factors into user and contextual requirements. We then engaged design team members through storyboarding, scenarios of use, and personas to inform the collaborative specification of NA-SB delivery. To facilitate this discussion, we divided NA-SB delivery into six segments: (1) young adult receives and completes needs assessment, (2) young adult “turns in” needs assessment, (3) data is documented, (4) data is interpreted to identify appropriate services/resources, (5) service/resource providers are notified, and (6) services and resources are provided. We then walked workshop attendees through each segment, priming them with the user and contextual requirements relevant to that segment. Together, we specified each segment of delivery, discussing both a pilot scenario as well as future broader implementation. The selected specification options were then vetted in terms of personas and scenarios of use generated from ethnographic data.

During the second design team workshop, we also discussed the future implementation of NA-SB, anticipating barriers and facilitators to implementation and brainstorming strategies to optimize NA-SB implementation. This discussion was informed by a list of barriers and facilitators gleaned from usability testing and ethnographic data. We used PollEverywhere to rank this list of barriers from most to least salient. We then discussed the three barriers ranked as the most salient in terms of the mechanisms driving those barriers as well as potential strategies to address them.

##### Analysis

EH took detailed notes during design team workshop #2 to capture all discussion points leading to decisions on NA-SB delivery and implementation; the meeting was also recorded for further elaboration on meeting notes. We synthesized and analyzed notes inductively to document the results of design team prototyping and generate guidance for NA-SB delivery and implementation.

## Results

By ensuring NA-SB directly responded to features of users and context, we designed NA-SB *for implementation*, potentially minimizing the strategies needed to address misalignment that may have otherwise existed. Furthermore, we designed NA-SB *for scale-up*; by engaging users from other cancer programs across the country to identify points of contextual variation which would require flexibility in delivery, we created a tool not overly tailored to one unique context. To allow for a more detailed focus on our methods, we have summarized our results in Additional File 5. This file includes results from usability testing (i.e., participant demographics, ratings of CNQ-YP needs, evaluation of CNQ-YP, and grouping of needs from concept mapping), ethnographic contextual inquiry (i.e., participant demographics, guided tour, and interview translation tables), and design team prototyping workshops (e.g., summaries of item decisions, selected concept mapping cluster map, anticipated implementations strategies). Briefly, the methods described above culminated in an NA-SB prototype, including a redesigned patient-reported outcome measure and referral pathways that are triggered based on needs young adults report, as well as a plan for implementation. To achieve our NA-SB prototype, many adaptations were made to the CNQ-YP to promote its usability and usefulness; for example, we added missing content (e.g., items on sexual health), removed unactionable content (i.e., needs that are not addressable by services), revised confusing or unpalatable items, streamlined the tool’s sequencing and response options, and, importantly, linked the needs assessed by the tool to referrals pathways expected to address them. In addition to adapting the CNQ-YP, we also identified context modifications needed to promote receptivity to NA-SB implementation (e.g., changes in social worker workflow to accommodate NA-SB administration; modification of electronic medical record to allow for documentation of NA-SB). To address remaining gaps between NA-SB and its implementation context that were not addressed by intervention or context modifications, we anticipated needed implementation strategies (e.g., building buy-in among providers across disease groups; taking a phased-in approach to implementation).

## Discussion

Increasingly, we have seen critiques of the traditional research pipeline which “spans from basic science to treatment development to efficacy and occasional effectiveness trials and then to implementation” [71]. From our perspective, we need to move away from a strict adherence to this supposed linear research trajectory. There is a need to embed implementation research earlier in the pipeline and expand the scope of the field to address critical gaps that are slowing the uptake of evidence (e.g., designing interventions that are implementable; obtaining a rich understanding of context and using that understanding to improve implementation and sustainment). Such efforts may be enhanced through the application of methods from other disciplines like UCD.

Just as embroidering requires compatible thread, fabric, and needle, implementation may be optimized by harmonizing EBP, context, and implementation strategies. We acknowledge that this analogy is imperfect; for example, some might regard embroidery as decoration or embellishment; on the contrary, our intention with this analogy is to convey the integration of the thread such that it becomes a part of the fabric itself. Despite its imperfection, the analogy is useful as it urges implementation scientists to attend equally to features of EBPs, context, and implementation strategies. Doing so has the potential to limit the challenges associated with complex EBPs and implementation strategies that burden stakeholders. In this study, we leveraged methods from UCD to harmonize EBP, context, and implementation; the benefits and challenges associated with these methods are summarized in Table 3.

**Table 3:** Benefits and challenges associated with UCD methods

Method	Description	Benefits	Challenges
Usability testing	Products or services are evaluated by testing them among potential users. Participants must represent real users and the researcher watches them complete representative tasks.	• Identifies usability and usefulness issues with EBP at any point in the development • Provides valuable source data for design team prototyping workshops • Can be done with a small number of participants	• Making decisions about who counts as a user and which individuals represent users more broadly • Prioritizing divergent feedback from different user groups • Requires multiple iterations to use it effectively
Ethnographic contextual inquiry	*Contextual inquiry*: in-depth data on a few carefully selected individuals informs a fuller understanding of users and their context. *Ethnography*: immersive analytic descriptions of behaviors that characterize and distinguish groups, including the knowledge and beliefs that generate and help interpret those behaviors.	• Elicits in-depth data on users, their tasks, and their context • Particularly helpful for understanding the multilevel, non-rational, or difficult-to-quantify contextual processes influencing implementation and sustainment • Sheds light on the differences between what people say and what people do • Provides valuable source data for design team prototyping workshops	• Can be time-intensive • Large amounts of data generated can be cumbersome to analyze and interpret • Requires the researcher to be nimble as they move through the participant’s context without being overly intrusive • Participants must be sampled carefully so as not to sacrifice all breadth of information for depth • Can position the researcher in difficult or emotionally charged situations, bringing us face-to-face with the hardships faced by the populations we study
Design team prototyping workshop	A multidisciplinary group of prospective users and other stakeholders convene to generate design solutions based on project data (e.g., usability testing and contextual inquiry data).	• Engages users in analysis to promote a shared understanding of the context • Provides platform and methods (e.g., translation tables, storyboards, personas, scenario of use) for translating contextual data into EBP adaptations, context modifications, and implementation strategies • Builds buy-in among prospective users	• Presenting project data in a way that is digestible to design team members • Weighing the importance of user feedback with the feasibility of design solutions • Inexpert application of UCD methods may lead to “feature creep,” in which new ideas are incorporated into the EBP without careful consideration and evaluation of the effects of the added features

In this case example, *usability testing* elicited user concerns about the CNQ-YP that may have limited its uptake in practice, allowing our design team to redesign the CNQ-YP to maximize usability and usefulness. For example, through concept mapping, providers identified needs assessed by the CNQ-YP which, as originally written, could not be addressed with available services/resources (e.g., “I feel frustrated”); assessing such unactionable needs would have produced additional burden for users, without improving care. Through the survey and cognitive interviews, young adults identified important missing content (e.g., sexual health), and other areas in which the CNQ-YP’s content, length, wording, and response format were unacceptable. By addressing these usability and usefulness concerns upfront, we designed a tool to be more feasible, acceptable, and appropriate to users.

Considering EBP characteristics like usability and usefulness in a vacuum may compromise implementation and burden stakeholders. To avoid these concerns, we leveraged UCD *contextual inquiry* methods to describe both NA-SB’s specific implementation context (i.e., NCCH) as well as its broader future scale-up context (i.e., other young adult cancer programs in the USA). To explore the context, UCD offers frameworks (e.g., Maguire’s framework), as well as questionnaires (e.g., System Usability Scale [29]), and a menu of methods (e.g., diary keeping, user surveys [26]) compatible with others used by implementation scientists in the assessment of implementation determinants. Despite some overlap in UCD and implementation science methods, UCD goes further than traditional barriers/facilitators assessment by embedding users more deeply in the process. In this case example, we used *ethnographic* contextual inquiry to obtain a detailed understanding of users and context. Additionally, we went further than traditional barriers/facilitators assessments by engaging users in analysis to promote a shared understanding of the context: our design team reviewed ethnography findings to ensure that the user interpretation of context remained central, as opposed to relying solely on the researcher’s interpretation of contextual data.

UCD also provides methods for translating *user and contextual factors* into *user and contextual requirements—*i.e., usability and usefulness determinants [26]. Translating contextual factors into contextual requirements using UCD *requirements engineering* approaches (e.g., translation tables, personas, and scenarios-of-use) could help implementation scientists prioritize implementation determinants by focusing attention on the critical subset of contextual factors that influence EBP usability and usefulness [17]. In this case example, the ethnography provided valuable source data for workshop materials, helping us to leverage design team expertise to identify these usability determinants and prioritize contextual features to target with EBP redesign, context preparation, or implementation strategies. For example, during design team workshop #2, we presented several alternative scenarios of use, or simple descriptions of plausible user interactions with NA-SB, to inform the specification of NA-SB delivery. These scenarios provide user- and task-oriented information about the context in which an EBP has to operate [72], and also offer concrete examples for design team members to react to. For example, scenarios of use helped our design team walk through different patient visit types (e.g., just infusion versus infusion + clinical visit) to ensure that design decisions about staffing and timing for NA-SB administration suited the range of potential appointments.

We used UCD to enhance the usability and usefulness of NA-SB and reduce the number of implementation strategies needed to embed the tool in routine care. However, where EBP and context diverge, UCD can help tailor strategies which make EBP and context more compatible. In this case example, we anticipated the areas where NA-SB provision may clash with user or contextual requirements, some of which could not be addressed by EBP redesign or context preparation. For example, NA-SB—a tool that spans across multiple domains of care—will require the cooperation of multiple departments and disciplines; although users are more likely to buy into a usable and useful tool [73, 74] and engaging users in its development likely generated some buy-in, additional implementation strategies targeting cross-department buy-in may be required. These remaining gaps in EBP-context fit inform the selection of strategies to promote NA-SB implementation. Leveraging UCD to identify the user and contextual requirements and tailor implementation strategies addresses an articulated need in the field [18, 75] and complement approaches for selecting and tailoring strategies that have recently been proposed in the implementation science literature [17]. Future work will assess the extent to which UCD minimizes the need for complex implementation strategies or, when needed, aids in the tailoring of strategies that are contextually appropriate and minimally burdensome.

As demonstrated by this case example, UCD can help implementation scientists to operationalize the field’s commitment to stakeholder engagement. For example, establishing a design team upfront ensured that users remained central throughout NA-SB development and implementation planning. Design team members offered key insights to inform data collection (e.g., review of instruments), data analysis (e.g., selection of concept mapping cluster map; prioritization of user and contextual requirements), and, ultimately, NA-SB and implementation strategy design. Further, design team members proved critical to the recruitment of users for usability testing and ethnographic data collection. UCD also offers methods for translating user feedback into design decisions, addressing another articulated gap in implementation science [76]. For example, the use of storyboards, personas, and scenarios of use allowed our design team to translate ethnographic data into NA-SB design features in a way that group discussion without such engagement methods may not have. Finally, UCD demands an iterative approach to user engagement, often with the same group of users reviewing prototypes at multiple time points; this type of iteration may be a key moderator in the relationship between stakeholder engagement and improved EBP design [50]. In keeping with the iterative nature of UCD, in future work, NA-SB will undergo additional refinement based on user interactions with our NA-SB prototype.

Applying UCD to implementation science has notable challenges. Embedding the extensive engagement UCD requires can sometimes be costly and time-intensive. Additionally, this level of engagement places issues of sampling and recruitment at the forefront. For example, the UCD process hinges on complex decisions about who counts as a user and which individuals accurately represent users more broadly. Prioritizing divergent feedback from multiple user groups [77], or weighing the relative importance of user feedback with the feasibility of design solutions, may not always be straightforward. Inexpert application of UCD methods may lead to “feature creep,” in which new ideas are incorporated into the EBP without careful consideration and evaluation of the effects of the added features. UCD’s emphasis on iterative design thinking and local insights may also raise concerns about diminishing fidelity as EBPs are recurrently revised to better align with context outside of the controlled environment where the EBP was originally designed and tested. Finally, implementation scientists may struggle to shoulder the challenges associated with incorporating new disciplines into already multidisciplinary teams and projects (e.g., reconciling terminology and frameworks). However, if implementation scientists are to leverage key insights from other disciplines, we must continue to surmount such roadblocks to knowledge integration.

## Conclusions

Implementing change in dynamic healthcare settings is complex; understanding the nuances of implementation requires a multimodal, multidisciplinary purview. To this end, implementation scientists have borrowed knowledge and approaches from systems science [78, 79], organizational studies [80], cultural adaptation [81], community-based participatory research [82], behavioral psychology [83], and quality improvement [84], just to name a few. We argue that UCD methods like usability testing, ethnographic contextual inquiry, and design team prototyping can join the list of approaches available to implementation scientists. This may first require investigation of where UCD and implementation science converge and diverge. Fortunately, efforts to this effect are currently underway [85]. While points of divergence may represent barriers to integration of the two fields, they may also represent important new insights and approaches for implementation scientists to consider.

Just as embroidery requires the alignment of thread, fabric, and needle, EBP implementation and sustainment requires harmonizing EBP, context, and implementation strategies. The importance of each of these has been acknowledged; however, methods for understanding the dynamic interplay among them and optimizing each *with respect* to the other two are lacking. UCD offers methods and approaches for achieving this. Future research should explore the utility of collaborating with UCD experts or embedding UCD approaches in implementation research [85]. In particular, we argue that UCD’s potential for promoting harmonization among EBP, context, and implementation should be tested empirically, work that is currently underway [86]. To the extent that UCD helps facilitate this harmonization, it will advance us toward the field’s goal of bridging the gap between research and practice.

## Supplementary Information

**Additional file 1.** This file contains the survey instrument used for the online survey of young adults, including demographic questions, questions from the Cancer Needs Questionnaire-Young People tool, and questions surrounding the tool’s usability and usefulness.

**Additional file 2.** This file contains the cognitive interview guide, including the original Cancer Needs Questionnaire-Young People.

**Additional file 3.** This file contains Maguire et al.’s framework of user and contextual factors to consider in User-Centered Design. The file also includes example questions within each domain of Maguire et al.’s framework used during ethnographic contextual inquiry (i.e., guided tours and semi-structured interviews).

**Additional file 4.** This file contains the Standards for Reporting Qualitative Research (SRQR) checklist.

**Additional file 5.** This file contains a summary of results from each phase of our user-centered design process. This includes results from usability testing (i.e., participant demographics, ratings of CNQ-YP needs, evaluation of CNQ-YP, and grouping of needs through concept mapping), ethnographic contextual inquiry (i.e., participant demographics, guided tour and interview translation tables), and design team prototyping workshops (e.g., summaries of item decisions, selected concept mapping cluster map, anticipated implementations strategies).

## Data Availability

Data collection instruments are available as additional files.

## Abbreviations

EBP: Evidence-based practice
UCD: User-centered design
NA-SB: Needs Assessment and Service Bridge
CNQ-YP: Cancer Needs Questionnaire - Young People
CSGM: Concept Systems GlobalMax©
