# Supplementary material for: Harmonizing evidence-based practice, implementation context, and implementation strategies with user-centered design: a case example in young adult cancer care
Source: Implement Sci Commun. 2021 Apr 26;2:45. doi: 10.1186/s43058-021-00147-4 (PMC8077816; doi:10.1186/s43058-021-00147-4)
Supplement: Supplementary file 1 — Additional file 1. This file contains the survey instrument used for the online survey of young adults, including demographic questions, questions from the Cancer Needs Questionnaire-Young People tool, and questions surrounding the tool’s usability and usefulness. [file 43058_2021_147_MOESM1_ESM.docx]

**Additional File 1. Young adult survey**

**Section 1: Demographics**

1. How do you describe your sex and gender identity? *(mark all that apply)*
   1. *Female*
   2. *Male*
   3. *Cisgender*
   4. *Genderqueer*
   5. *Intersex*
   6. *Transgender*
   7. *A gender not listed ___________*
2. How old are you?
   1. *Scroll bar 18-30*
3. How long has it been since you were diagnosed with cancer?
   1. *Less than 3 months*
   2. *Between 3 and 6 months*
   3. *Between 7 and 12 months*
   4. *Greater than 12 months*
4. Are you currently in active treatment?
   1. *Yes*
   2. *No*
5. Please select the type of cancer you were diagnosed with from the list below.
   1. *Non-Hodgkin Lymphoma*
   2. *Hodgkin Lymphoma*
   3. *Leukemia*
   4. *Sarcoma*
   5. *Cervical*
   6. *Other female reproductive*
   7. *Male reproductive*
   8. *Thyroid*
   9. *Brain*
   10. *Melanoma*
   11. *Colorectal*
   12. *Breast*
   13. *Other (please indicate): ________*
6. Select the stage of your cancer at diagnosis.
   1. *0*
   2. *I/II*
   3. *III/IV*
   4. *Unknown/unstaged*
7. With which racial/ethnic group do you most identify?
   1. *Hispanic (all races)*
   2. *Non-Hispanic American Indian/ Alaska Native*
   3. *Non-Hispanic Asian or Pacific Islander*
   4. *Non-Hispanic Black*
   5. *Non-Hispanic White*
   6. *Other/unknown*
8. Who do you live with? *Check all that apply.*
   1. *Parent(s)*
   2. *Spouse*
   3. *Non-spouse partner*
   4. *Child/children*
   5. *Roommate(s) (not parent, spouse, or child)*
   6. *I live alone*
   7. *Other*
9. What is your education level?
   1. *Less than high school diploma*
   2. *Completed high school*
   3. *Some college/vocational training*
   4. *Associate degree*
   5. *College graduate*
   6. *Graduate degree or some post-graduate education*
10. What is your insurance source?
    1. *Self-pay*
    2. *No insurance*
    3. *Employer/school*
    4. *Spouse’s employer/school*
    5. *Parent*
    6. *Medicare*
    7. *Medicaid*
    8. *Military/TRICARE*
    9. *Other*

**Section 2 : CNQ-YP**

We are now going to ask you to complete a survey tool that was developed in Australia to assess the needs of young adults with cancer, called the **Cancer Needs Questionnaire- Young People (CNQ-YP).**

As you complete the CNQ-YP, imagine that your doctor has asked you to complete this survey either during or before an appointment with them, as part of your clinical care. After you have completed the CNQ-YP, we will ask you just a few more questions about what you thought about it.

| **No Need** | All my needs were met for this issue or this was not a problem for me. |
| --- | --- |
| **Low Need** | I needed a low amount of help with this problem but was not able to get it. |
| **Moderate Need** | I needed a moderate amount of help with this problem but was not able to get it. |
| **High Need** | I needed a high amount of help with this problem but was not able to get it. |
| **Very High Need** | I needed a very high amount of help with this problem but was not able to get it. |

| The following questions ask about any needs you may have had **at any time** **since your cancer diagnosis.** |
| --- |

**1. Treatment Environment and Care**

| **I had the following needs…** | | | | | | |
| --- | --- | --- | --- | --- | --- | --- |
| **BEFORE TREATMENT** | | | | | | |
| **Cancer treatment staff telling me:** | | **No Need** | **Low Need** | **Moderate Need** | **High Need** | **Very High Need** |
| **1** | about my diagnosis | ○ | ○ | ○ | ○ | ○ |
| **2** | what might happen during treatment | ○ | ○ | ○ | ○ | ○ |
| **3** | whether I had the option to decline treatment | ○ | ○ | ○ | ○ | ○ |
| **4** | about the short term side-effects of treatment | ○ | ○ | ○ | ○ | ○ |
| **5** | about the long term side-effects of treatment | ○ | ○ | ○ | ○ | ○ |
| **6** | my chances of a full recovery | ○ | ○ | ○ | ○ | ○ |
| **7** | what would happen when treatment finished | ○ | ○ | ○ | ○ | ○ |
| **8** | whether I would be able to have children | ○ | ○ | ○ | ○ | ○ |
| **DURING TREATMENT** | | | | | | |
| **Cancer treatment staff telling me:** | | **No Need** | **Low Need** | **Moderate Need** | **High Need** | **Very High Need** |
| **9** | whether my treatment was working | ○ | ○ | ○ | ○ | ○ |
| **10** | my test results as soon as possible | ○ | ○ | ○ | ○ | ○ |
| **11** | the way I felt was normal | ○ | ○ | ○ | ○ | ○ |
| **Being able to have:** | | **No Need** | **Low Need** | **Moderate Need** | **High Need** | **Very High Need** |
| **12** | have time to myself | ○ | ○ | ○ | ○ | ○ |

| **I had the following needs…** | | | | | | |
| --- | --- | --- | --- | --- | --- | --- |
| **AFTER TREATMENT** | | | | | | |
| **Cancer treatment staff telling me:** | | **No Need** | **Low Need** | **Moderate Need** | **High Need** | **Very High Need** |
| **13** | how to manage my medication | ○ | ○ | ○ | ○ | ○ |
| **14** | what I could do to stay healthy | ○ | ○ | ○ | ○ | ○ |
| **15** | what to do if I noticed a particular side-effect | ○ | ○ | ○ | ○ | ○ |
| **THROUGHOUT TREATMENT** | | | | | | |
| **Having cancer treatment staff who:** | | **No Need** | **Low Need** | **Moderate Need** | **High Need** | **Very High Need** |
| **16** | listened to my concerns | ○ | ○ | ○ | ○ | ○ |
| **17** | treated me as an individual | ○ | ○ | ○ | ○ | ○ |
| **18** | were respectful | ○ | ○ | ○ | ○ | ○ |
| **19** | were approachable | ○ | ○ | ○ | ○ | ○ |
| **20** | were friendly | ○ | ○ | ○ | ○ | ○ |
| **21** | could have a laugh with me | ○ | ○ | ○ | ○ | ○ |
| **22** | explained what they were doing | ○ | ○ | ○ | ○ | ○ |
| **23** | spoke to me in a way that I could understand | ○ | ○ | ○ | ○ | ○ |
| **24** | let me talk about my feelings | ○ | ○ | ○ | ○ | ○ |
| **25** | let me ask questions | ○ | ○ | ○ | ○ | ○ |
| **26** | let me make decisions about my treatment | ○ | ○ | ○ | ○ | ○ |
| **27** | talked to me in private, without my family | ○ | ○ | ○ | ○ | ○ |

| **I had the following needs…** | | | | | | |
| --- | --- | --- | --- | --- | --- | --- |
| **AT THE CANCER TREATMENT CENTRE** | | | | | | |
| **Being able to have:** | | **No Need** | **Low Need** | **Moderate Need** | **High Need** | **Very High Need** |
| **28** | privacy | ○ | ○ | ○ | ○ | ○ |
| **29** | pleasant surroundings | ○ | ○ | ○ | ○ | ○ |
| **30** | good food | ○ | ○ | ○ | ○ | ○ |
| **31** | a choice of cancer specialists | ○ | ○ | ○ | ○ | ○ |
| **32** | the same cancer treatment staff throughout treatment | ○ | ○ | ○ | ○ | ○ |
| **33** | a choice of times for appointments | ○ | ○ | ○ | ○ | ○ |

**2. Education**

| **Since my cancer diagnosis, I have had problems enrolling at:** *(please choose as many as apply)* | | | | | | |
| --- | --- | --- | --- | --- | --- | --- |
| **S1** | - school - TAFE - university/college - other place of study *(please write)*_____________________________________________ - none of the above | | | | | |
| **Since my cancer diagnosis, I have attended:** *(please choose as many as apply)* | | | | | | |
| **S2** | - school - TAFE - university/college - other place of study *(please write)*_____________________________________________ - none of the above *(go to Question S3)* | | | | | |
| **I had the following needs…** | | | | | | |
| **WHEN STUDYING** | | | | | | |
| **Being able to:** | | **No Need** | **Low Need** | **Moderate Need** | **High Need** | **Very High Need** |
| **34** | attend classes | ○ | ○ | ○ | ○ | ○ |
| **35** | get extensions/special consideration | ○ | ○ | ○ | ○ | ○ |
| **36** | get guidance about study options or future career paths | ○ | ○ | ○ | ○ | ○ |

**3. Work**

| **Since my cancer diagnosis, I have had problems finding work:** *(please choose as many as apply)* | | | | | | |
| --- | --- | --- | --- | --- | --- | --- |
| **S3** | - full-time - part-time/casual - unpaid voluntary work - other type of work *(please write)*______________________________________________ - none of the above | | | | | |
| **Since my cancer diagnosis, I have been employed:** *(please choose as many as apply)* | | | | | | |
| **S4** | - full-time - part-time/casual - unpaid voluntary work - other type of work *(please write)*______________________________________________ - none of the above (*go to Question 40)* | | | | | |
| **I had the following needs…** | | | | | | |
| **WHEN EMPLOYED** | | | | | | |
| **Knowing:** | | **No Need** | **Low Need** | **Moderate Need** | **High Need** | **Very High Need** |
| **37** | how much work I would miss | ○ | ○ | ○ | ○ | ○ |
| **38** | how to ask managers/co-workers for support | ○ | ○ | ○ | ○ | ○ |
| **39** | that managers/co-workers had support to help them cope with my situation | ○ | ○ | ○ | ○ | ○ |

**4. Information and Activities**

| **I had the following needs…** | | | | | | |
| --- | --- | --- | --- | --- | --- | --- |
| **DURING TREATMENT** | | | | | | |
| **Being able to:** | | **No Need** | **Low Need** | **Moderate Need** | **High Need** | **Very High Need** |
| **40** | spend time with people my own age | ○ | ○ | ○ | ○ | ○ |
| **41** | talk to people my age who had been through a similar experience | ○ | ○ | ○ | ○ | ○ |
| **AT THE CANCER TREATMENT CENTRE** | | | | | | |
| **Being able to have:** | | **No Need** | **Low Need** | **Moderate Need** | **High Need** | **Very High Need** |
| **42** | leisure spaces and activities | ○ | ○ | ○ | ○ | ○ |
| **SINCE MY CANCER DIAGNOSIS** | | | | | | |
| **Finding information that:** | | **No Need** | **Low Need** | **Moderate Need** | **High Need** | **Very High Need** |
| **43** | was specifically designed for me | ○ | ○ | ○ | ○ | ○ |
| **44** | described relaxation techniques | ○ | ○ | ○ | ○ | ○ |

| The next group of questions ask about any needs you may have had **in the last month.**  *We realise that your needs may have changed during different stages of your cancer experience. Please only tell us about needs you have had in the last month. If you have not had any needs in the last month, please select ‘No Need’.* |
| --- |

**5. Feelings and Relationships**

| **I had the following needs…** | | | | | | |
| --- | --- | --- | --- | --- | --- | --- |
| **IN THE LAST MONTH** | | | | | | |
| **Feeling:** | | **No Need** | **Low Need** | **Moderate Need** | **High Need** | **Very High Need** |
| **45** | frustrated | ○ | ○ | ○ | ○ | ○ |
| **46** | anxious or nervous | ○ | ○ | ○ | ○ | ○ |
| **IN THE LAST MONTH** | | | | | | |
| **Worrying about:** | | **No Need** | **Low Need** | **Moderate Need** | **High Need** | **Very High Need** |
| **47** | my cancer spreading | ○ | ○ | ○ | ○ | ○ |
| **48** | my cancer returning | ○ | ○ | ○ | ○ | ○ |
| **49** | whether my cancer treatment has worked | ○ | ○ | ○ | ○ | ○ |
| **50** | having cancer treatment | ○ | ○ | ○ | ○ | ○ |
| **51** | how my family is coping | ○ | ○ | ○ | ○ | ○ |
| **IN THE LAST MONTH** | | | | | | |
| **Finding:** | | **No Need** | **Low Need** | **Moderate Need** | **High Need** | **Very High Need** |
| **52** | inner strength | ○ | ○ | ○ | ○ | ○ |
| **IN THE LAST MONTH** | | | | | | |
| **Being able to:** | | **No Need** | **Low Need** | **Moderate Need** | **High Need** | **Very High Need** |
| **53** | accept my diagnosis | ○ | ○ | ○ | ○ | ○ |
| **54** | be independent | ○ | ○ | ○ | ○ | ○ |

| **S5** | **Do you have:**  *(please choose as many as apply)*   - a spouse/partner or boyfriend/girlfriend *(please answer Question 55)* - sibling/s or step-brothers/sisters *(please answer Questions 56-58)* - none of the above *(go to Question 59)* | | | | | |
| --- | --- | --- | --- | --- | --- | --- |
| **I had the following needs…** | | | | | | |
| **IN THE LAST MONTH** | | | | | | |
| **Coping with:** | | **No Need** | **Low Need** | **Moderate Need** | **High Need** | **Very High Need** |
| **55** | changes in my relationship with my partner | ○ | ○ | ○ | ○ | ○ |
| **IN THE LAST MONTH** | | | | | | |
| **Coping with:** | | **No Need** | **Low Need** | **Moderate Need** | **High Need** | **Very High Need** |
| **56** | changes in my relationships with my sibling/s | ○ | ○ | ○ | ○ | ○ |
| **IN THE LAST MONTH** | | | | | | |
| **Knowing how to:** | | **No Need** | **Low Need** | **Moderate Need** | **High Need** | **Very High Need** |
| **57** | ask my sibling/s for support | ○ | ○ | ○ | ○ | ○ |
| **58** | give support to my sibling/s | ○ | ○ | ○ | ○ | ○ |

**6. Daily Life**

| **I had the following needs…** | | | | | | |
| --- | --- | --- | --- | --- | --- | --- |
| **IN THE LAST MONTH** | | | | | | |
| **Being able to:** | | **No Need** | **Low Need** | **Moderate Need** | **High Need** | **Very High Need** |
| **59** | make plans or think about the future | **○** | ○ | ○ | ○ | ○ |
| **IN THE LAST MONTH** | | | | | | |
| **Coping with:** | | **No Need** | **Low Need** | **Moderate Need** | **High Need** | **Very High Need** |
| **60** | changes in my physical ability | **○** | ○ | ○ | ○ | ○ |
| **61** | changes in my appearance | **○** | ○ | ○ | ○ | ○ |
| **62** | not being able to do the same things as other people my age | **○** | ○ | ○ | ○ | ○ |
| **63** | my parent/s being overprotective | **○** | ○ | ○ | ○ | ○ |
| **IN THE LAST MONTH** | | | | | | |
| **Managing:** | | **No Need** | **Low Need** | **Moderate Need** | **High Need** | **Very High Need** |
| **64** | pain | ○ | ○ | ○ | ○ | ○ |
| **65** | medication | ○ | ○ | ○ | ○ | ○ |
| **66** | physical side effects of treatment | ○ | ○ | ○ | ○ | ○ |
| **67** | feeling tired | ○ | ○ | ○ | ○ | ○ |
| **68** | loss of mobility | ○ | ○ | ○ | ○ | ○ |
| **69** | to take part in social activities | ○ | ○ | ○ | ○ | ○ |
| **70** | to travel to social events | ○ | ○ | ○ | ○ | ○ |

**Section 3: Usability Testing**

You have completed the CNQ-YP. Now, we will ask you some questions about your experience filling out the CNQ-YP. As you are answering these questions, imagine that your doctor has asked you to complete the CNQ-YP either during or before an appointment with them, as part of your clinical care.

|  | Completely disagree (1) | Disagree (2) | Neither agree nor disagree (3) | Agree (4) | Completely agree (5) |
| --- | --- | --- | --- | --- | --- |
| The CNQ-YP meets my approval. |  |  |  |  |  |
| The CNQ-YP is appealing to me. |  |  |  |  |  |
| I like the CNQ-YP. |  |  |  |  |  |
| I welcome the CNQ-YP. |  |  |  |  |  |

Display This Question:

If Acceptability = The CNQ-YP meets my approval. [ Completely disagree ]

And Acceptability = The CNQ-YP meets my approval. [ Disagree ]

And Acceptability = The CNQ-YP is appealing to me. [ Completely disagree ]

And Acceptability = The CNQ-YP is appealing to me. [ Disagree ]

And Acceptability = I like the CNQ-YP. [ Completely disagree ]

And Acceptability = I like the CNQ-YP. [ Disagree ]

And Acceptability = I welcome the CNQ-YP. [ Completely disagree ]

And Acceptability = I welcome the CNQ-YP. [ Disagree ]

Please explain what you dislike about the CNQ-YP:

|  | Completely disagree (1) | Disagree (2) | Neither agree nor disagree (3) | Agree (4) | Completely agree (5) |
| --- | --- | --- | --- | --- | --- |
| The CNQ-YP seems fitting as a needs assessment tool for adolescents and young adults with cancer. |  |  |  |  |  |
| The CNQ-YP seems like a suitable tool for my doctor to administer to me as part of my cancer care. |  |  |  |  |  |
| The CNQ-YP seems applicable to adolescent and young adult cancer care. |  |  |  |  |  |
| The CNQ-YP seems like a good match for adolescents and young adults. |  |  |  |  |  |

Display This Question:

If Appropriateness = The CNQ-YP seems fitting as a needs assessment tool for adolescents and young adults with cancer. [ Completely disagree ]

And Appropriateness = The CNQ-YP seems fitting as a needs assessment tool for adolescents and young adults with cancer. [ Disagree ]

And Appropriateness = The CNQ-YP seems like a suitable tool for my doctor to administer to me as part of my cancer care. [ Completely disagree ]

And Appropriateness = The CNQ-YP seems like a suitable tool for my doctor to administer to me as part of my cancer care. [ Disagree ]

And Appropriateness = The CNQ-YP seems applicable to adolescent and young adult cancer care. [ Completely disagree ]

And Appropriateness = The CNQ-YP seems applicable to adolescent and young adult cancer care. [ Disagree ]

And Appropriateness = The CNQ-YP seems like a good match for adolescents and young adults. [ Completely disagree ]

And Appropriateness = The CNQ-YP seems like a good match for adolescents and young adults. [ Disagree ]

Please describe your concerns about the appropriateness of the CNQ-YP for adolescent and young adult cancer care.

|  | Completely disagree (1) | | Disagree (2) | | Neither agree nor disagree (3) | | Agree (4) | Completely agree (5) |
| --- | --- | --- | --- | --- | --- | --- | --- | --- |
| The CNQ-YP seems like something my doctor could administer to all of their patients |  |  | |  | |  | |  |
| The CNQ-YP seems possible for me to complete as part of my care |  |  | |  | |  | |  |
| The CNQ-YP seems doable. |  |  | |  | |  | |  |
| The CNQ-YP seems easy to use. |  |  | |  | |  | |  |

Display This Question:

If Feasibility = The CNQ-YP seems like something my doctor could administer to all of their patients [ Completely disagree ]

And Feasibility = The CNQ-YP seems like something my doctor could administer to all of their patients [ Disagree ]

And Feasibility = The CNQ-YP seems possible for me to complete as part of my care [ Completely disagree ]

And Feasibility = The CNQ-YP seems possible for me to complete as part of my care [ Disagree ]

And Feasibility = The CNQ-YP seems doable. [ Completely disagree ]

And Feasibility = The CNQ-YP seems doable. [ Disagree ]

And Feasibility = The CNQ-YP seems easy to use. [ Completely disagree ]

And Feasibility = The CNQ-YP seems easy to use. [ Disagree ]

Please explain your concerns about the feasibility of implementing the CNQ-YP in your cancer treatment center:

|  | Completely disagree (1) | Disagree (2) | Neither agree nor disagree (3) | Agree (4) | Completely agree (5) |
| --- | --- | --- | --- | --- | --- |
| My doctors would gain a good understanding of my needs from reviewing my answers to this survey. |  |  |  |  |  |
| I would consider using services/resources offered by my doctor if they matched them to the needs I identified in this survey. |  |  |  |  |  |

Display This Question:

If Actionability = My doctors would gain a good understanding of my needs from reviewing my answers to this survey. [ Completely diagree ]

And Actionability = My doctors would gain a good understanding of my needs from reviewing my answers to this survey. [ Disagree ]

Please explain why your concerns about the CNQ-YP as a picture of your needs at any given time

Display This Question:

If Actionability = I would consider using services/resources offered by my doctor if they matched them to the needs I identified in this survey. [ Completely diagree ]

And Actionability = I would consider using services/resources offered by my doctor if they matched them to the needs I identified in this survey. [ Disagree ]

Please explain why you may not consider using services/resources offered to you by your doctor based on needs identified in the CNQ-YP

Are there any important needs which aren't captured in the CNQ-YP?

- yes *(please list them here)* ________________________________________________
- No

Is there anything in the CNQ-YP that shouldn't be?

- yes *(please indicate here):* ________________________________________________
- no

If you have any other thoughts about the CNQ-YP, please indicate them below:
