# Supplementary material for: Harmonizing evidence-based practice, implementation context, and implementation strategies with user-centered design: a case example in young adult cancer care
Source: Implement Sci Commun. 2021 Apr 26;2:45. doi: 10.1186/s43058-021-00147-4 (PMC8077816; doi:10.1186/s43058-021-00147-4)
Supplement: Supplementary file 2 — Additional file 2. This file contains the cognitive interview guide, including the original Cancer Needs Questionnaire-Young People. [file 43058_2021_147_MOESM2_ESM.docx]

**Additional File 2. Cognitive Interview Guide**

**INTRODUCTION**

Thanks for agreeing to speak with me. We are developing a user-friendly tool to assess the needs of adolescents and young adults with cancer. This tool would be used by your cancer care providers to figure out what services and resources they should direct you to.

As a starting point for developing this tool, we are using the Cancer Needs Questionnaire-Young People (or CNQ-YP), which you may remember from the online survey you completed a few weeks ago. The purpose of today is to have you provide input on the CNQ-YP, and identify areas where it may need improvement. We are also interested in getting your thoughts about how this tool might be delivered in practice.

This interview might be a little different from others you’ve done. We are less interested in your answers to CNQ-YP questions and more interested in learning how usable the CNQ-YP is.  We would like to know how you arrive at your answers and find out if any questions or response options are confusing or weird to you. There are no right or wrong answers to any of these questions.

For each question, what I would like you to do is read it aloud and then, please tell me what the question means in your own words. Then, please “think aloud” as you formulate your response. By “think aloud” I mean verbalize your thought process as you interpret each question, recall the information you need to respond, look through the response options, and decide your response.

When you are done “thinking aloud” I will ask you a series of questions about the CNQ-YP. Your participation is completely voluntary and you may skip any question. Please feel free to criticize this tool openly- I will not be offended. Your thoughts and comments about the tool are very important. This interview will last approximately 60 minutes. Do you have any questions for me before we begin?

During the interview, I will be taking notes. Since it is difficult to write as fast as people talk, I would like to audio record this discussion, as well. If at any time you would like me to stop audio recording, just tell me, and I will do so. The tapes will only be heard by the study team working on this project. Once we have used the tapes to make sure that my notes are accurate, the tapes will be destroyed.

Can we begin?

Before I ask you to look at the tool, I would like to ask you a “warm up” question to introduce you to the think-aloud process. I would like for you to visualize the windows in the place you live. As you count up how many windows you have, tell me what you are seeing and thinking about.

**Example questions**

1. Please tell me in your own words what this question means. Then proceed to “think aloud” as you formulate your response.
2. Are there any words here that seem ambiguous, or confusing?
   1. Which ones?
   2. How is the word ambiguous or confusing?
   3. What did you think the word meant?
3. Do you have the information in your memory to respond to this question?
   1. If yes, “think aloud” as you access this information in your memory?
   2. If no, who would have this information? How easy or difficult would it be to gather it?
4. Does this seem to you like an important question?
   1. How important would it be to get accurate information for this question?
5. How did you arrive at your answers?
   1. How easy or difficult is it to identify your most accurate response?
   2. Can you suggest any changes that would make it easier to identify your most accurate response?
6. Is there anything else about this question that you want to mention?
7. Are the choices of response adequate to capture your lived experience?
   1. If not, what choices would you like to see included?
8. Is the organization of the questions easy to follow?
   1. If not, how can the questions be best grouped together?
9. Are there additional questions you might add to this questionnaire to address needs that are not captured by the survey in its current form?
