# Supplementary material for: Harmonizing evidence-based practice, implementation context, and implementation strategies with user-centered design: a case example in young adult cancer care
Source: Implement Sci Commun. 2021 Apr 26;2:45. doi: 10.1186/s43058-021-00147-4 (PMC8077816; doi:10.1186/s43058-021-00147-4)
Supplement: Supplementary file 3 — Additional file 3. This file contains Maguire et al.’s framework of user and contextual factors to consider in User-Centered Design. The file also includes example questions within each domain of Maguire et al.’s framework used during ethnographic contextual inquiry (i.e., guided tours and semi-structured interviews). [file 43058_2021_147_MOESM3_ESM.docx]

**Additional File 3. Framework for contextual inquiry**

| **User group characteristics** | |
| --- | --- |
| **Factors** | **Example questions** |
| - User type - User role - Goals for intervention - Perceived benefits of intervention - Perceived costs of intervention - Experience with intervention - Related experience - Task knowledge - Organizational knowledge - Training - Input device skills - Qualifications | **Providers/Staff**   - What is your role in caring for AYAs with cancer? - What experience do you have with cancer needs assessment tools or asking AYAs about their needs? - Are you familiar with the services and resources available at your institution for AYAs?   **AYAs**   - Have you ever been asked to complete survey that asks you about your needs? - Do you think - What do you think the benefits of this intervention would be? - What do you think the costs of this intervention would be? |
| **User tasks** | |
| **Factors** | **Example questions** |
| - Task list   - Task 1   - Task 2   - Etc. - For each task:   - Task characteristics   - Task goal/output   - Task steps   - Task frequency   - Task duration   - Task flexibility   - Task dependencies   - Task output   - Risks resulting from error   - Critical demands | **Providers/Staff**   - Walk me through your daily patient care/ administrative tasks.   - What are the goals of each task?   - How long does each take?   - How often is each performed? - Given your current task load, would you be able to administer a needs assessment to all AYAs?   - When? As part of which task?   **AYAs**   - Walk me what a typical appointment looks like. - Would completing a survey be possible during any of these tasks? |
| **Technical and physical environment** | |
| **Factors** | **Example questions** |
| - Hardware - Software - Network - Reference materials - Other equipment - Physical environment - Space - Location | **Providers/Staff**   - Where do you currently record information about needs expressed by AYAs?   - In the electronic health record?   - Is this information easy to find? - What kind of reference materials do you give to patients? - What kind of referral network does your institution have for AYAs?   **AYAs**   - Where do you currently seek information about resources and services available for AYAs? - Where do you wait before your appointments? - Do you typically open appointment reminders sent by email? - What kind of resources have you been given during appointments? |
| **Organizational environment** | |
| **Factors** | **Example questions** |
| - Structure - Group working - Work practices - Assistance - Interruptions - Management structure - Communications structure - Attitudes and culture - Computer use policies - Organization aims - Industrial relations - Job design - Job functions - Hours of work - Job flexibility - Performance monitoring and feedback | **Providers/Staff**   - Does your institution support the establishment of systems or processes specifically for AYAs? - Who is in charge of AYA cancer care? - How do you communicate with other providers in your institution? - What information are you required to enter in patients’ chart? - Has your institution implemented needs assessments for other patient groups?   - If yes, how has that gone? - What barriers do you anticipate to implementing AYA NA-SB in your organization?   - Staffing?   Whose job is it to coordinate care for AYAs? |

1. Maguire M. Methods to support human-centred design. *International Journal of Human-Computer Studies.* 2001;55(4):587-634.
