# Supplementary material for: Harmonizing evidence-based practice, implementation context, and implementation strategies with user-centered design: a case example in young adult cancer care
Source: Implement Sci Commun. 2021 Apr 26;2:45. doi: 10.1186/s43058-021-00147-4 (PMC8077816; doi:10.1186/s43058-021-00147-4)
Supplement: Supplementary file 4 — Additional file 4. This file contains the Standards for Reporting Qualitative Research (SRQR) checklist. [file 43058_2021_147_MOESM4_ESM.docx]

**Additional File 4. Standards for Reporting Qualitative Research (SRQR)**

| **Title and abstract** | | |
| --- | --- | --- |
| S1 | Title | Harmonizing evidence-based practice, implementation context, and implementation strategies with user-centered design: a case example in young adult cancer care |
| S2 | Abstract | ***Background.*** Attempting to implement evidence-based practices in contexts for which they are not well-suited may compromise their fidelity and effectiveness or burden users (e.g., patients, providers, healthcare organizations) with elaborate strategies intended to force implementation. To improve the fit between evidence-based practices and contexts, implementation science experts have called for methods for adapting evidence-based practices and contexts, and tailoring implementation strategies; yet, methods for considering the dynamic interplay among evidence-based practices, contexts, and implementation strategies remain lacking. We argue that harmonizing the three can be accomplished with User-Centered Design, an iterative and highly stakeholder-engaged set of principles and methods.  ***Methods.*** This paper presents a case example in which we used User-Centered Design methods and a three-phase User-Centered Design process to design a care coordination intervention for young adults with cancer. Specifically, we used *usability testing* to redesign an existing evidence-based practice (i.e., patient-reported outcome measure that served as the basis for intervention) to optimize usability and usefulness, an *ethnographic user and contextual inquiry* to prepare the context (i.e., comprehensive cancer center) to promote receptivity to implementation, and iterative *prototyping workshops with a multidisciplinary design team* to design the care coordination intervention and anticipate implementation strategies needed to enhance contextual fit.  ***Results.*** Our User-Centered Design process resulted in the Young Adult Needs Assessment and Service Bridge (NA-SB), including a patient-reported outcome measure redesigned to promote usability and usefulness and a protocol for its implementation. By ensuring NA-SB directly responded to features of users and context, we designed NA-SB *for implementation*, potentially minimizing the strategies needed to address misalignment that may have otherwise existed. Furthermore, we designed NA-SB *for scale-up*; by engaging users from other cancer programs across the country to identify points of contextual variation which would require flexibility in delivery, we created a tool not overly tailored to one unique context.  ***Conclusions.*** User-Centered Design can help maximize usability and usefulness when designing evidence-based practices, preparing contexts, and informing implementation strategies- in effect, harmonizing evidence-based practices, contexts, and implementation strategies to promote implementation and effectiveness. |
| **Introduction** | | |
| S3 | Problem formulation | Each year, more than 20,000 young adults between the ages of 18 and 30 are diagnosed with cancer[[25](#_ENREF_25)]; many of them do not receive services to meet the range of needs they experience during and after cancer treatment[[26-30](#_ENREF_26)]. Young adults’ unmet needs result in negative outcomes, including higher distress[[27](#_ENREF_27), [28](#_ENREF_28)], poorer health-related quality of life[[31](#_ENREF_31)], and higher physical symptom burden[[26](#_ENREF_26)]. Despite the complexity and scope of their needs, young adults often do not use potentially beneficial services/resources, even when access is not an issue[[32-34](#_ENREF_32)]. This disconnect between young adult needs and their use of existing services/resources suggests the need for a care coordination model that (1) effectively assesses young adults’ multifaceted, age-specific, individual, and dynamic needs, and (2) uses that information to efficiently connect them to services/resources.  A substantial step toward this care coordination model was the development of the first multidimensional measure of unmet needs designed specifically for adolescents and young adults: the Cancer Needs Questionnaire - Young People (CNQ-YP)[[35](#_ENREF_35), [36](#_ENREF_36)]. However, limitations to the usability and usefulness of patient-reported outcome measures like the CNQ-YP (e.g., length; wording ambiguity; redundancy or missing content; lack of connection between identified needs and follow-up actions) have frustrated their real-world implementation and effect on patient outcomes[[37](#_ENREF_37), [38](#_ENREF_38)]. |
| S4 | Purpose or research question | In this project, we used User-Centered Design to redesign the CNQ-YP to optimize its usability and usefulness and prepare for its implementation in the University of North Carolina’s Lineberger Comprehensive Cancer Center. Our UCD process produced the Needs Assessment and Service Bridge (NA-SB), a care coordination intervention for young adults with cancer. |
| **Methods** | | |
| S5 | Qualitative approach and research paradigm | To gather detailed information about prospective users and the context for NA-SB implementation, we used ethnography (i.e., guided tours and semi-structured interviews), a promising yet underused method for implementation research[[50](#_ENREF_50)]. By documenting naturally-occurring user tasks and interactions among patients and providers through in-depth observation, ethnography provides rich data on implementation context[[51](#_ENREF_51), [52](#_ENREF_52)]. Ethnographic methods are relevant to UCD because they offer more nuanced understanding of users and context than traditional questionnaires or interviews, including novel insights on user tasks, attitudes, and interactions with their environment[[17](#_ENREF_17), [21](#_ENREF_21), [53](#_ENREF_53)]. We used a novel UCD user and contextual factor framework[[21](#_ENREF_21)] to guide data collection and analysis, combining *ethnography* and *contextual inquiry*. |
| S6 | Researcher characteristics and reflexivity | EH, who conducted ethnographic data collection, is a young adult and has been personally affected by cancer but has never been a cancer patient or provider. Thus, although she understands developmental issues pertinent to the young adult population, she lacks understanding of the experience of these issues colliding with a cancer diagnosis. Through ethnographic data collection, she sought to learn more about this experience and the role of the health care system in supporting young adults through it. It is also worth noting that EH is white, cisgender, and non-disabled and thus, has a different perspective than young adult participants who did not fall within these privileged categories. |
| S7 | Context | We leveraged UCD *contextual inquiry* methods to describe both NA-SB’s specific implementation context (i.e., Lineberger) as well as its broader future scale-up context (i.e., other young adult cancer programs in the United States). |
| S8 | Sampling strategy | To capture the perspective of potential NA-SB implementers, we conducted guided tours with our clinical partners at Lineberger (n=2). To capture the patient perspective, we conducted guided tours with young adults ages 18-30 receiving inpatient or outpatient care at Lineberger (n=10). Our clinical partners at Lineberger facilitated the recruitment of young adults for guided tours by distributing a recruitment flyer and connecting EH via email to those interested.  We conducted semi-structured interviews with the leaders of young adult programs and advocacy groups identified by our clinical partners through their professional network, who had previously facilitated survey and concept mapping recruitment: program managers (n=2), nurse navigators (n=2), and patient navigators (n=2) serving primarily young adults. |
| S9 | Ethical issues pertaining to human subjects | This study was approved by the University of North Carolina’s Institutional Review Board (19-0255). |
| S10 | Data collection methods | EH conducted four-hour guided tours with clinical partners as they completed clinical, administrative and other duties, asking questions about their tasks and thoughts. EH followed young adults and accompanying family members from the moment they entered the hospital for their outpatient appointments until the moment they exited, asking them questions as they interacted with their environment and healthcare professionals, while attempting to minimize participant disruptions. For inpatient guided tours, EH spent two hours with young adults receiving inpatient care. EH took extensive field notes and audio-recorded portions of the guided tours for which only consenting parties were present. We offered young adult participants a $50 participation incentive.  EH conducted one-hour semi-structured telephone interviews. At the end of each interview, EH summarized major takeaways for member checking[[47](#_ENREF_47)]. We audio-recorded and transcribed interviews verbatim. |
| S11 | Data collection instruments and technologies | To promote the flexibility required for guided tours[[55](#_ENREF_55), [56](#_ENREF_56)], we identified potential questions based on four domains of Maguire et al.’s typology of user and contextual factors to consider in UCD from which we could choose: (1) user characteristics, (2) user tasks, (3) physical and technical environment, and (4) organizational environment[[21](#_ENREF_21)] (**Additional File 3**).  With input from the design team, we developed a semi-structured interview guide based on Maguire’s typology[[21](#_ENREF_21)] and guided tour findings. |
| S12 | Units of study | we conducted guided tours with our clinical partners at Lineberger (n=2). To capture the patient perspective, we conducted guided tours with young adults ages 18-30 receiving inpatient or outpatient care at Lineberger (n=10).  We conducted semi-structured interviews with the leaders of young adult programs and advocacy groups who had previously facilitated survey and concept mapping recruitment: program managers (n=2), nurse navigators (n=2), and patient navigators (n=2) serving primarily young adults. |
| S13 | Data processing | During guided tours, EH took extensive field notes and audio-recorded portions of the guided tours for which only consenting parties were present.  We audio-recorded and transcribed interviews verbatim. |
| S14 | Data analysis | We used template analysis, identifying *a priori* themes based on Maguire’s constructs and allowing for identification of additional themes[[46](#_ENREF_46)]. Two study authors independently coded excerpts from guided tour field notes and interview transcriptions per Maguire constructs. For each domain, they collaboratively synthesized user and contextual factors and created a “translation table”[[57](#_ENREF_57)], which translated factors into their implications for NA-SB design and implementation. For example, providers reported the importance of integrating new tools into the electronic medical record; we translated this into the requirement that NA-SB interface with Lineberger’s electronic medical record. All requirements were vetted and prioritized by the design team during the second workshop (see description below). |
| S15 | Techniques to enhance trustworthiness | Two study authors independently coded excerpts from guided tour field notes and interview transcriptions per Maguire constructs.  At the end of each interview, EH summarized major takeaways for member checking[[47](#_ENREF_47)]. |
| **Results/Findings** | | |
| S16 | Synthesis and interpretation | To allow for more detailed focus on our methods, we have limited our reporting of results to key takeaways on how UCD may enable EBP-context-implementation strategy harmonization. More detailed results will be presented in future publications. Briefly, though, the methods described above culminated in an NA-SB prototype, including a redesigned patient-reported outcome measure and a protocol for its implementation. By ensuring NA-SB directly responded to features of users and context, we designed NA-SB *for implementation*, potentially minimizing the strategies needed to address misalignment that may have otherwise existed. Furthermore, we designed NA-SB *for scale-up*; by engaging users from other cancer programs across the country to identify points of contextual variation which would require flexibility in delivery, we created a tool not overly tailored to one unique context. |
| S17 | Links to empirical data | To allow for more detailed focus on our methods, we have limited our reporting of results to key takeaways on how UCD may enable EBP-context-implementation strategy harmonization. |
| **Discussion** | | |
| S18 | Integration with prior work, implications, transferability, and contributions to the field | We used UCD to enhance the usability and usefulness of NA-SB and reduce the number of implementation strategies needed to embed the tool in routine care. By addressing usability and usefulness concerns upfront, we designed a tool to be more feasible, acceptable, and appropriate to users.  To explore context, UCD offers frameworks such (e.g., Maguire’s framework), as well as questionnaires (e.g., System Usability Scale[[59](#_ENREF_59)]), and a menu of methods (e.g., diary keeping, user surveys, etc.[[22](#_ENREF_22)]) compatible with others used by implementation scientists in the assessment of implementation determinants. Despite some overlap in UCD and implementation science methods, UCD goes further than traditional barriers/facilitators assessment by embedding users more deeply in the process.  Leveraging UCD to identify user and contextual requirements and tailor implementation strategies addresses an articulated need in the field[[15](#_ENREF_15), [63](#_ENREF_63)] and complements approaches for selecting and tailoring strategies that have recently been proposed in the implementation science literature[[14](#_ENREF_14)].  As demonstrated by this case example, UCD can help implementation scientists to operationalize the field’s commitment to stakeholder engagement.  Just as embroidery requires the alignment of thread, fabric, and needle, EBP implementation and sustainment requires harmonizing EBP, context, and implementation strategies. The importance of each of these has been acknowledged; however, methods for understanding the dynamic interplay among them and optimizing each *with respect* to the other two are lacking. UCD offers methods and approaches for achieving this. |
| S19 | Limitations | Applying UCD to implementation science has notable challenges. Embedding the extensive engagement UCD requires can sometimes be costly and time intensive. Additionally, this level of engagement places issues of sampling and recruitment at the forefront. For example, the UCD process hinges on complex decisions about who counts as a user and which individuals accurately represent users more broadly. Prioritizing divergent feedback from multiple user groups[[65](#_ENREF_65)], or weighing the relative importance of user feedback with the feasibility of design solutions, may not always be straightforward. Inexpert application of UCD methods may lead to ‘feature creep,’ in which new ideas are incorporated into the EBP without careful consideration and evaluation of the effects of the added features. UCD’s emphasis on iterative design thinking and local insights may also raise concerns about diminishing fidelity as EBPs are recurrently revised to better align with context outside of the controlled environment where the EBP was originally designed and tested. Finally, implementation scientists may struggle to shoulder the challenges associated with incorporating new disciplines into already multidisciplinary teams and projects (e.g., reconciling terminology and frameworks). In addition to such practical differences, there may be fundamental philosophical differences between the two fields. In general, implementation science focuses more-so on the EBP and UCD on the users; where there is divergence between what is best for the EBP and what is best for the user, reconciling these competing viewpoints may be difficult. However, if implementation scientists are to leverage key insights from other disciplines, they must continue to surmount such roadblocks to knowledge integration. |
| **Other** | | |
| S20 | Conflicts of interest | The authors declare that they have no competing interests. |
| S21 | Funding | Dr. Haines’ effort was supported by funding from UNC Lineberger’s University Cancer Research Fund and by 2T32 CA122061 from the National Cancer Institute. Dr. Dopp is an investigator with the Implementation Research Institute, at the George Warren Brown School of Social Work, Washington University in St. Louis; through an award from the National Institutes of Mental Health (5R25MH08091607) and the Department of Veterans Affairs, Health Services Research & Development Service, Quality Enhancement Research Initiative (QUERI). Dr. Birken’s effort was supported by the National Center for Advancing Translational Sciences, National Institutes of Health, through Grant KL2TR002490. The content is solely the responsibility of the authors and does not necessarily represent the official views of the NIH. |
