# Supplementary material for: Harmonizing evidence-based practice, implementation context, and implementation strategies with user-centered design: a case example in young adult cancer care
Source: Implement Sci Commun. 2021 Apr 26;2:45. doi: 10.1186/s43058-021-00147-4 (PMC8077816; doi:10.1186/s43058-021-00147-4)
Supplement: Supplementary file 5 — Additional file 5. This file contains a summary of results from each phase of our user-centered design process. This includes results from usability testing (i.e., participant demographics, ratings of CNQ-YP needs, evaluation of CNQ-YP, and grouping of needs through concept mapping), ethnographic contextual inquiry (i.e., participant demographics, guided tour and interview translation tables), and design team prototyping workshops (e.g., summaries of item decisions, selected concept mapping cluster map, anticipated implementations strategies). [file 43058_2021_147_MOESM5_ESM.docx]

**Additional File 5**

**Summary of results**

**1. Usability testing results**

1.1 Survey

**Table 1. Survey participants**

| **Sex and gender identity** |  |
| --- | --- |
| Female | 55 (78.6%) |
| Male | 15 (21.4%) |
| Transgender | 2 (2.9%) |
| **Age** |  |
| Mean (SD) | 24.24 (3.96) |
| **Race** |  |
| Hispanic (all races) | 10 (14.3%) |
| Non-Hispanic American Indian/ Alaska Native | 3 (4.3%) |
| Non-Hispanic Asian or Pacific Islander | 2 (2.9%) |
| Non-Hispanic Black | 1 (1.4%) |
| Non-Hispanic White | 49 (70.0%) |
| Other | 5 (7.1%) |
| **Cancer type** |  |
| Non-Hodgkin lymphoma | 5 (7.1%) |
| Hodgkin lymphoma | 15 (21.4%) |
| Leukemia | 11 (15.7%) |
| Sarcoma | 9 (12.9%) |
| Cervical | 1 (1.4%) |
| Other female reproductive | 3 (4.3%) |
| Male reproductive | 1 (1.4%) |
| Thyroid | 5 (7.1%) |
| Brain | 5 (7.1%) |
| Melanoma | 2 (2.9%) |
| Colorectal | 1 (1.4%) |
| Breast | 4 (5.7%) |
| Other | 8 (11.4%) |
| **Stage at diagnosis** |  |
| 0 | 1 (1.4%) |
| I/II | 24 (34.3%) |
| III/IV | 22 (31.4%) |
| Unknown/ unstaged | 23 (32.9%) |
| **Time since diagnosis** |  |
| < 3 months | 3 (4.3%) |
| 3-6 months | 9 (12.9%) |
| 7-12 months | 8 (11.4%) |
| >12 months | 50 (71.4%) |
| **In active treatment?** |  |
| no | 51 (72.9%) |
| yes | 19 (27.1%) |
| **Cohabitants** |  |
| Parent(s) | 29 (42.7%) |
| Spouse | 12 (17.7%) |
| Non-spouse partner | 7 (10.3%) |
| Child/children | 4 (5.9%) |
| Roommate(s) (not parent, spouse, or child) | 12 (17.7%) |
| Lives alone | 8 (11.8%) |
| **Education level** |  |
| < high school | 2 (2.9%) |
| Completed high school | 9 (13.2%) |
| Some college/ vocational training | 23 (33.8%) |
| Associate degree | 3 (4.4%) |
| College graduate | 15 (22.1%) |
| Graduate degree or some post-graduate education | 16 (23.5%) |
| **Insurance source** |  |
| Self-pay | 4 (5.9%) |
| No insurance | 3 (4.4%) |
| Employer/ school | 18 (26.5%) |
| Spouse’s employer/ school | 2 (2.9%) |
| Parent | 25 (36.8%) |
| Medicare | 3 (4.4%) |
| Medicaid | 8 (11.8%) |
| Military/ TRICARE | 8 (11.8%) |
| other | 5 (7.3%) |
|  | N=70 |

Figure 1. Survey respondents’ average rating of needs: CNQ-YP ‘treatment environment & care’ section

**
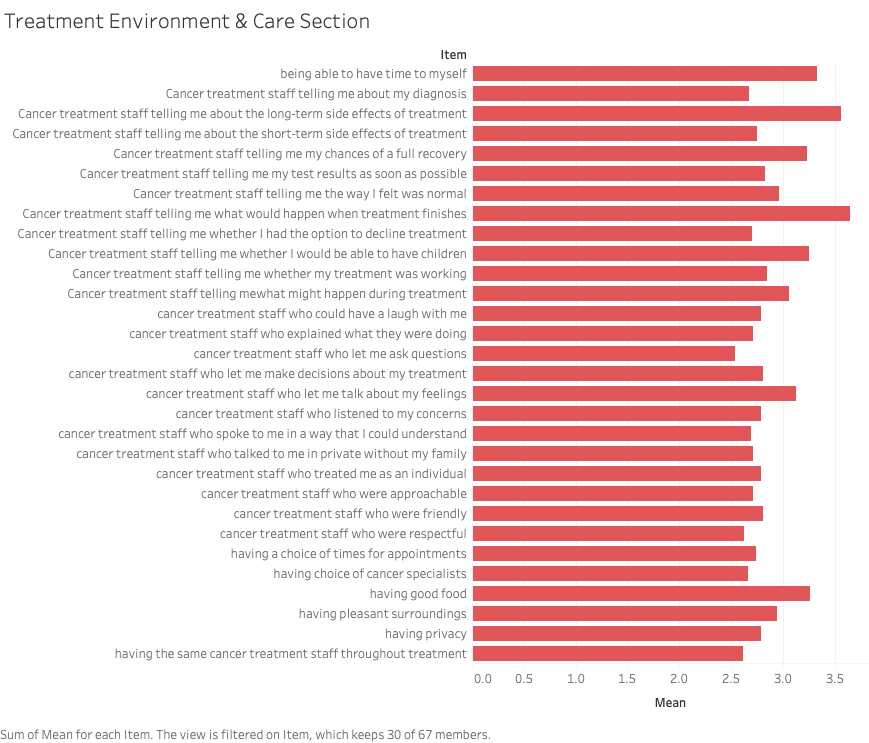
**

***** Respondents rated needs a scale of 1-5 (1=no need; 5=very high need)

Figure 2. Survey respondents’ average rating of needs: CNQ-YP ‘education’ section

**
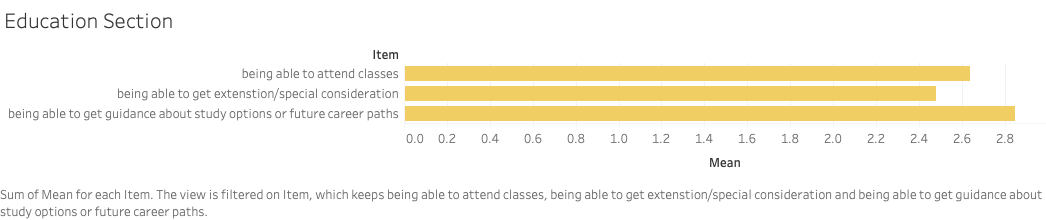
**

***** Respondents rated needs a scale of 1-5 (1=no need; 5=very high need)

Figure 3. Survey respondents’ average rating of needs: CNQ-YP ‘work’ section

**
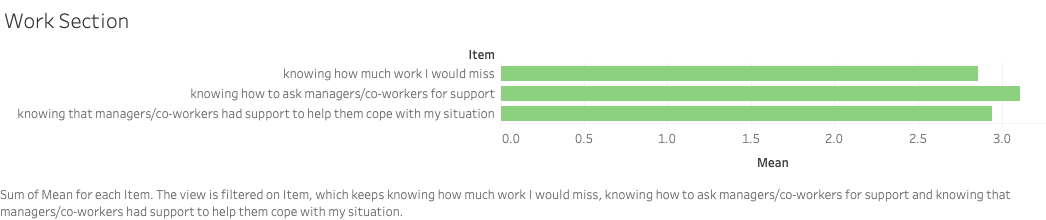
**

***** Respondents rated needs a scale of 1-5 (1=no need; 5=very high need)

Figure 4. Survey respondents’ average rating of needs: CNQ-YP ‘feelings & relationships’ section

**
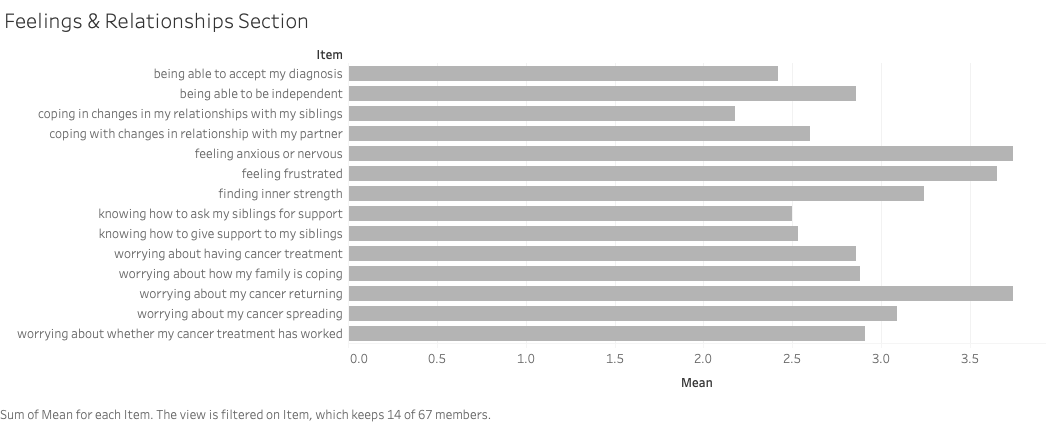
**

***** Respondents rated needs a scale of 1-5 (1=no need; 5=very high need)

Figure 5. Survey respondents’ average rating of needs: CNQ-YP ‘daily life’ section


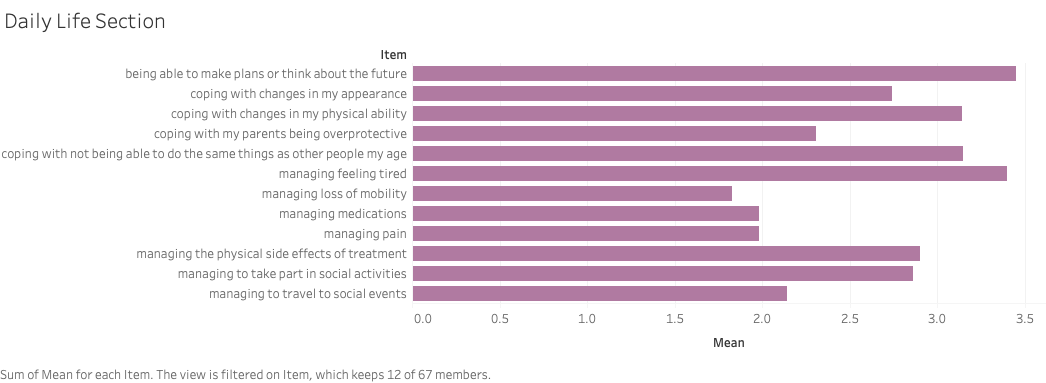


***** Respondents rated needs a scale of 1-5 (1=no need; 5=very high need)

**Table 2. Survey respondents’ evaluation of the CNQ-YP**

| **Measure** | **Statistics** |
| --- | --- |
| **Acceptability** |  |
| The CNQ-YP meets my approval | 4.0 (0.7) |
| The CNQ-YP is appealing to me | 3.8 (0.9) |
| I like the CNQ-YP | 3.7 (0.9) |
| I welcome the CNQ-YP | 3.8 (0.8) |
| **Index** | 3.8 (0.7) |
| **Appropriateness** |  |
| The CNQ-YP seems fitting as a needs assessment tool for adolescents and young adults with cancer | 3.9 (0.9) |
| The CNQ-YP seems like a suitable tool for my doctor to administer to me as part of my cancer care | 4.1 (0.6) |
| The CNQ-YP seems applicable to adolescent and young adult cancer care | 4.2 (0.6) |
| The CNQ-YP seems like a good match for adolescents and young adults | 4.1 (0.6) |
| **Index** | 4.1 (0.6) |
| **Feasibility** |  |
| The CNQ-YP seems like something my doctor could administer to all of their patients | 4.0 (0.8) |
| The CNQ-YP seems possible for me to complete as part of my care | 4.1 (0.6) |
| The CNQ-YP seems doable | 4.1 (0.6) |
| The CNQ-YP seems easy to use | 3.9 (0.9) |
| **Index** | 4.0 (0.6) |
| **Actionability** |  |
| My doctors would gain a good understanding of my needs from reviewing my answers to this survey | 4.2 (0.7) |
| I would consider using services/resources offered by my doctor if they matched them to the needs I identified in this survey | 4.4 (0.6) |
| **Index** | 4.3 (0.6) |

**Table 3. Survey respondents’ feedback on CNQ-YP content**

| **Is there anything missing from the CNQ-YP?** | |
| --- | --- |
| no | 60% |
| yes | 40%  financial needs, sexual health, mental health, social needs, physical activity, in-hospital space and activities specifically for AYAs, need for specialists (e.g., nutritionists, physical therapists) |
| **Is there anything in the CNQ-YP that shouldn’t be?** | |
| no | 97% |
| yes | 3%  Cumbersome response scales and item response format |

1.2 Cognitive Interviews

**Table 4. Suggestions for CNQ-YP refinement from cognitive interviews and changes made in response**

| **Category** | **Theme** | **Changes made** |
| --- | --- | --- |
| **Response format** | Confusion surrounding “no need” response option including both no need, and need already met | Changed the name of the “no need” response option to “no need/need met” |
|  | Item S1 and S2 (“since my cancer diagnosis I have enrolled at/had problems enrolling at”) response option “TAFE”(i.e., “technical and further education”) not understood | Removed response option “TAFE” from S1 and S2 |
|  | For items S1 and S2 (“since my cancer diagnosis I have enrolled at/had problems enrolling at”), AYAs wanted a response option related to internships | Added response option for S1 and S2: “internship (paid or unpaid)” |
|  | In Items S3 and S4 (“since my cancer diagnoses I have been employed/ have had problems finding work”) response option “part-time/casual”, AYAs didn’t like the term “casual” | Removed the word “casual” so response option just reads “part-time” |
|  | For Items S3 and S4 (“since my cancer diagnoses I have been employed/ have had problems finding work”), AYAs wanted a response option for trade school/apprenticeship | Added response option for S3 and S4: “trade school or apprenticeship” |
| **Question format** | Throughout the CNQ-YP the questions are broken up into different rows. For example, “I had the following need”, “before treatment”, “cancer treatment staff telling me”, and “about my diagnosis” are on separate lines. AYAs found this confusing. | All question wording consolidated onto one line. |
| **Lookback periods** | In general, AYAs found the multiple lookback periods (e.g., “before treatment”, “during treatment”, “after treatment”, etc.) confusing. Although they emphasized that needs do change depending on how far along you are in your treatment trajectory, these lookback periods were not considered helpful for informing current service provision | All needs items were anchored to needs currently being experienced (i.e., at the time of assessment completion). Redundancies stemming from the multiple lookback periods were removed. |
| **Treatment Environment and Care section** | Item 2 (“cancer treatment staff telling me what might happen during treatment”) interpreted the same as Item 4 (“about the short-term side effects of treatment”) | Removed Item 2 |
|  | Item 3 (“cancer treatment staff telling me whether I have the option to decline treatment”) perceived as less relevant/important | Removed Item 3 |
|  | AYAs did not like Item 6 (“cancer treatment staff telling me my chances of a full recovery”) because it is unclear what is meant by “full recovery” | Removed Item 6 |
|  | Section lacks item about sexual health | Added item assessing whether treatment staff provided information about sexual health |
|  | Item 9 (“cancer treatment staff telling me whether my treatment was working”) is too narrow | Changed item to “how my treatment is working” |
|  | Item 12 (“being able to have time to myself”) perceived as unimportant | Removed Item 12 |
|  | Item 14 (“cancer treatment staff telling me what I could do to stay healthy”) perceived as too vague; interpreted as putting the onus on AYAs to prevent secondary cancers which may be out of their control | Changed Item 14 to “cancer treatment staff giving me information about nutrition and exercise” |
|  | Item 16 (“having cancer treatment staff who listened to my concerns”) and Item 24 (“having cancer treatment staff who let me talk about my feelings”) perceived as redundant | Collapsed items 16 and 24 into “listened to my concerns and let me talk about my feelings” |
|  | Item 19 (“having cancer treatment staff who were approachable”) and Item 20 (“having cancer treatment staff who were friendly”) perceived as redundant | Collapsed Items 19 and 20 into “having cancer treatment staff who were friendly and approachable” |
|  | Item 22 (“having cancer treatment staff who explained what they were doing”) does not capture the importance of explaining *before* doing; redundant with Item 23 (“having cancer treatment staff who spoke to me in a way that I could understand”) | Collapsed Items 22 and 23 into “having cancer treatment staff who explained what they were doing before they did it, in a way that I could understand” |
| **Education section** | Items 34-36 had the question stem “I had the following needs when studying”. AYAs interpreted this as actively studying for a test. | Changed the question stem for Items 34-36 to “I had the following needs while enrolled at school” |
|  | Section is missing an item related to financial aid or loan repayment | Added item “being able to get guidance about financial aid or loan repayment options” |
| **Work section** | Item 39 (“knowing that managers/co-workers had support to help them cope with my situation”) not perceived as important | Removed Item 39 |
|  | Section lacks item about health insurance, which is a major need with respect to employment | Added item “worrying about my health insurance coverage” |
| **Information and Activities section** | AYAs did not understand/like Item 43 “finding information that was specifically designed for me” | Removed Item 43 |
| **Feelings and Relationships section** | Item 45 (“feeling frustrated”) not perceived as important relative to other psychosocial concerns | Removed Item 45 |
|  | Item 45 (“feeling anxious or nervous”) doesn’t fully capture the feeling of fear which is pervasive during treatment | Changed item 45 to “feeling anxious or scared” |
|  | Section lacking item about depression | Added item “feeling depressed” |
|  | Item 48 (“worrying about my cancer returning”) does not capture pervasive fear surrounding secondary cancers | Changed Item 48 to “worrying about my cancer returning or secondary cancers” |
|  | Item 50 (“worrying about having cancer treatment”) too vague | Removed Item 50 |
|  | Section only includes items assessing changes in relationship with partner and siblings; not inclusive of all the important relationships that may be affected by cancer | Added items assessing changes in relationship with parent/s and changes in relationships with friend/s |
| **Daily Life section** | Item 63 (“coping with my parent/s being overprotective”) not relevant for many AYAs | Changed Item 63 to “coping with my parent/s and/or partner being overprotective” |
|  | Section has item about physical side effects of treatment (Item 66) but none about the emotional side effects of treatment | Added item “managing emotional side effects of treatment” |
|  | Item 67 (“feeling tired”) does not capture fatigue, which AYAs felt was more severe than tiredness | Changed Item 67 to “feeling tired/fatigued” |
|  | AYAs did not understand or like Item 70 (“managing to travel to social event”) | Removed Item 70 |

1.3 Concept mapping

######

###### **Table 5. Concept mapping participants**

| **Location** |  |
| --- | --- |
| California | 5 |
| Florida | 2 |
| Illinois | 3 |
| Indiana | 1 |
| Michigan | 1 |
| Missouri | 1 |
| New York | 1 |
| North Carolina | 2 |
| Ohio | 2 |
| South Carolina | 1 |
| Tennessee | 2 |
| Texas | 4 |
| Utah | 1 |
| **Cancer Program Type** |  |
| NCI-designated comprehensive cancer center | 19 |
| Teaching hospital cancer program | 13 |
| Pediatric cancer program | 14 |
| Community cancer center | 3 |
| Hospital-based cancer program | 15 |
| Freestanding cancer center program | 1 |
| **Role** |  |
| Oncologist | 3 |
| Physician (non-oncology) | 1 |
| Nurse practitioner | 1 |
| Oncology nurse navigator | 3 |
| Nurse | 1 |
| Patient navigator | 1 |
| Social worker | 7 |
| Health educator | 1 |
| Other | 8 |
| **Years in role** | |
| Less than 5 | 16 |
| Between 5 and 10 | 5 |
| Between 10 and 20 | 2 |
| Greater than 20 | 3 |
| **Does your cancer program provide care specifically to adolescents and young adults?** | |
| yes | 24 |
| no | 2 |
|  | **N=26** |

###### **Table 6. Concept mapping participants’ average rating of each CNQ-YP need’s importance and actionability**

| **#** | **Statement** | **Importance Average** | **Actionability Average** |
| --- | --- | --- | --- |
| 1 | Cancer treatment staff telling me about my diagnosis | 5 | 5 |
| 2 | Cancer treatment staff telling me about the short-term side effects of treatment | 4.92 | 5 |
| 3 | Cancer treatment staff telling me about the long-term side effects of treatment | 4.84 | 4.8333 |
| 4 | Cancer treatment staff telling me what will happen when treatment finishes | 4.6 | 4.6667 |
| 5 | Cancer treatment staff telling me whether I will be able to have children | 4.84 | 4.5 |
| 6 | Cancer treatment staff telling me about how my treatment is working | 4.92 | 4.7391 |
| 7 | Cancer treatment staff telling me my test results as soon as possible | 4.44 | 4.4783 |
| 8 | Cancer treatment staff telling me the way I feel is normal | 4.36 | 4.5833 |
| 9 | Cancer treatment staff giving me information about sexual health | 4.52 | 4.5417 |
| 10 | Cancer treatment staff giving me information about nutrition and exercise. | 4.32 | 4.625 |
| 11 | Cancer treatment staff telling me what to do if I noticed a particular side effect | 4.92 | 4.9167 |
| 12 | Having cancer treatment staff who listened to my concerns and let me talk about my feelings | 4.68 | 4.5 |
| 13 | Having cancer treatment staff who treated me as an individual | 4.72 | 4.5833 |
| 14 | Having cancer treatment staff who were respectable | 4.6 | 4.4167 |
| 15 | Having cancer treatment staff who were approachable and friendly | 4.44 | 4.375 |
| 16 | Having cancer treatment staff who could have a laugh with me | 3.88 | 4.0435 |
| 17 | Having cancer treatment staff who explained what they are doing in a way I could understand | 4.72 | 4.4583 |
| 18 | Having cancer treatment staff who let me ask questions | 4.8333 | 4.7083 |
| 19 | Having cancer treatment staff who let me make decisions about my treatment | 4.8 | 4.5833 |
| 20 | Having cancer treatment staff who talked to me in private, without my family | 4.4 | 4.2917 |
| 21 | Being able to have privacy | 4.36 | 3.9583 |
| 22 | Being able to have pleasant surroundings | 3.8 | 3.25 |
| 23 | Being able to have good food | 3.6 | 3.2083 |
| 24 | Being able to have a choice of cancer care specialists | 4.12 | 3.0833 |
| 25 | Being able to have the same cancer treatment staff throughout treatment | 3.64 | 3.125 |
| 26 | Being able to have a choice of times for appointments | 3.56 | 3.0833 |
| 27 | Being able to attend classes (if enrolled in school) | 4.08 | 3.4583 |
| 28 | Being able to get extensions/special consideration (if enrolled in school) | 4.28 | 3.6667 |
| 29 | Knowing how much work I would miss | 4.24 | 3.4348 |
| 30 | Being able to get guidance about study options or future career paths | 3.84 | 3.9583 |
| 31 | Being able to get guidance about financial aid or loan repayment options | 4.24 | 4.2083 |
| 32 | Knowing how to ask managers/coworkers for support | 3.92 | 3.9167 |
| 33 | Worrying about my health insurance coverage | 4.72 | 4.1739 |
| 34 | Being able to spend time with people my own age | 4.6 | 4.25 |
| 35 | Being able to talk to people my own age who had been through a similar experience | 4.56 | 4.4583 |
| 36 | Being able to have leisure spaces and activities | 3.8 | 3.5833 |
| 37 | Finding information that described relaxation techniques (e.g., yoga, meditation) | 3.88 | 4.5833 |
| 38 | Feeling anxious or scared | 4.5417 | 4.3043 |
| 39 | Feeling depressed | 4.75 | 4.3478 |
| 40 | Worrying about my cancer spreading | 4.3333 | 3.913 |
| 41 | Worrying about my cancer returning or secondary cancers | 4.25 | 4 |
| 42 | Worrying about whether my cancer treatment has worked | 4.4167 | 3.9565 |
| 43 | Worrying about how my family is coping | 4.2917 | 4.0435 |
| 44 | Finding inner strength | 4.44 | 3.9167 |
| 45 | Being able to accept my diagnosis | 4.36 | 3.75 |
| 46 | Being able to be independent | 4.2 | 3.4583 |
| 47 | Coping with changes in my relationship to my partner | 4.6 | 3.9583 |
| 48 | Coping with changes in my relationship to my sibling/s | 4.25 | 3.9583 |
| 49 | Coping with changes in my relationship to my parent/s | 4.5 | 3.9583 |
| 50 | Coping with changes in my relationship to my friend/s | 4.4583 | 3.9167 |
| 51 | Being able to make plans or think about the future | 4.5 | 3.9583 |
| 52 | Coping with changes in my physical ability | 4.625 | 3.875 |
| 53 | Coping with changes in my appearance | 4.4583 | 3.75 |
| 54 | Coping with not being able to do the same things as other people my age | 4.4583 | 3.625 |
| 55 | Coping with my parent/s and/or partner being overprotective | 3.9167 | 3.7917 |
| 56 | Managing pain | 4.88 | 4.5417 |
| 57 | Managing my medications | 4.92 | 4.5 |
| 58 | Managing physical side effects of treatment | 4.8 | 4.3333 |
| 59 | Managing emotional side effects of treatment | 4.8 | 4.25 |
| 60 | Managing feeling tired/fatigued | 4.52 | 3.75 |
| 61 | Managing loss of mobility | 4.48 | 3.9167 |
| 62 | Managing to take part in social activities | 4.52 | 4.0417 |

**Figure 6. Concept mapping point map**


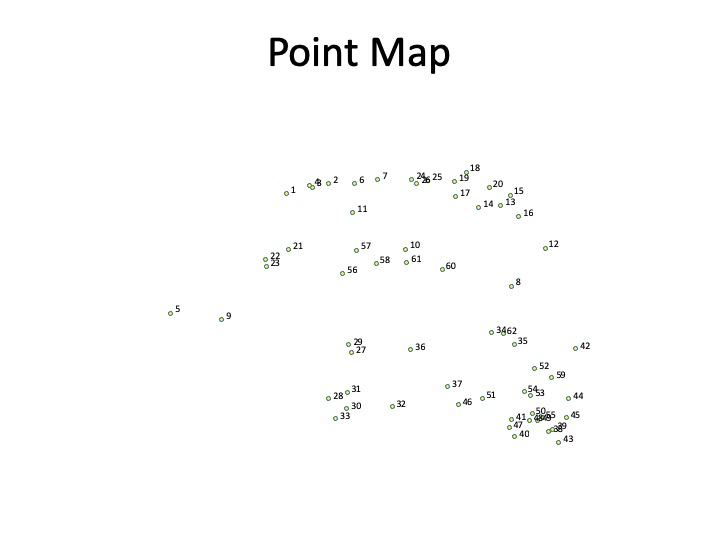


*See legend in **Table 6**

*Participants sorted and rated a set of 62 AYA needs. This point map displays the relationship and proximity of needs to one another. Each point on the map represents one AYA need. Those closer in proximity to each other were sorted together more often by concept mapping participants than those farther apart. The stress value for this point map was 0.2474, demonstrating goodness of fit. The lower the stress value, the better the fit between the map and the input matrix.

**2. Ethnographic contextual inquiry results**

2.1 Guided tours

**Table 7. AYA guided tour participants**

| **Sex and gender identity** |  |
| --- | --- |
| Female | 5 |
| Male | 3 |
| **Age** |  |
| Mean (SD) | 23.4 (5.0) |
| **Race** |  |
| Hispanic (all races) | 1 |
| Non-Hispanic Black | 2 |
| Asian | 1 |
| Non-Hispanic White | 4 |
| **Cancer type** |  |
| Non-Hodgkin Lymphoma | 1 |
| Hodgkin’s Lymphoma | 1 |
| Osteosarcoma | 2 |
| Chronic Myeloid Leukemia | 1 |
| Burkitt’s Lymphoma | 1 |
| Testicular cancer | 1 |
| Liver cancer | 1 |
| **Time since diagnosis** |  |
| < 3 months | 2 |
| 3-6 months | 2 |
| 7-12 months | 1 |
| >12 months | 3 |
| **Cohabitants** |  |
| Parent(s) | 2 |
| Spouse | 2 |
| Non-spouse partner | 3 |
| Child/children | 1 |
| Lives alone | 1 |
|  | N=8 |

**Table 8. Guided tour translation table**

| **Construct** | **Summary of contextual factors** | **NA-SB requirements** |
| --- | --- | --- |
| **Outer context** | | |
| **Patient characteristics** | - At NCCH, around 450 individuals ages 15-39 are diagnosed each year |  |
| Location | - Many AYAs do not live locally - Proximity to hospital is a major factor influencing AYAs’ use of services and resources | - Transportation represents an important barrier for NA-SB to address. - To the extent possible, NA-SB should be flexible to the diverse locations of AYAs. - Note: in the context of COVID-19, NCCH’s AYA program has developed much more flexibility with virtual visits; this presents new opportunities to administer NA-SB and render services virtually. |
| Technology | - AYAs are tech-savvy and use a broad range of technologies to manage their cancer | - Electronic delivery of NA-SB should be considered. |
| **Inner context** | | |
| **Structure** | - NCCH’s AYA program is relatively new (est. 2015) and currently undergoing expansion | - The expansion of NCCH’s AYA program represents an opportunity for the implementation of NA-SB. |
| **Staffing** | - LL (AYA program director/ social worker) and AS (AYA program medical director/ pediatric oncologist) work closely with non-AYA-specific providers and staff across disease groups from both pediatric and adult oncology, including oncologists, nurse practitioners, physician assistants, and other oncology providers. - NCCH’s model of cancer care hinges on providing multidisciplinary services and resources to support patients. | - NA-SB delivery should leverage AYA-specific staff where possible - NA-SB should facilitate the communication and sharing of information across providers from pediatric and adult oncology and across disease groups. - Clear referral pathways should be established with other providers to facilitate the provision of services from across departments and disciplines. |
| **Leadership** | - NCCH’s AYA program falls partially under the umbrella of the Comprehensive Cancer Support Program. | - NA-SB should leverage Comprehensive Cancer Support Program resources, where applicable |
| **Physical space** | - NCCH is positioned within a sprawling medical campus; AYAs’ appointments are held in disparate locations across the medical campus. - For AYAs receiving care in adult oncology, clinical appointments are held in separate disease group clinics which are positioned in disparate locations across NCCH. Adult oncology patients share an infusion space. - For AYAs receiving care in pediatric oncology, registration, labs, clinical appointments, and infusions occur in one centralized location on the first floor (the Pediatric Oncology Clinic). - NCCH is in the process of building an AYA-specific infusion center. | - NA-SB delivery should accommodate the variety of locations where AYAs have appointments. - The centralized layout of pediatric oncology may facilitate the communication and information sharing that NA-SB will require. - For AYAs receiving infusion treatments, the addition of an AYA-specific infusion space represents an opportunity for NA-SB service and resource provision, as AYAs will be concentrated in one central location. |
| **Culture** | - NCCH has a culture of collaboration and improvement but provider buy-in is an important prerequisite of any change initiative. | - The collaborative, improvement-oriented culture of NCCH bodes well for NA-SB implementation. Building buy-in among providers will be critical to implementation. |
| **Reference materials** | - AYAs did not find brochures and other non-tailored resources very helpful. | - The provision of reference materials through NA-SB should be highly tailored to the unique needs reported by each AYA on the needs assessment. |
| **Individuals** | | |
| **Program director/**  **social worker** | - LL divides her time among (1) administrative tasks, (2) research-related tasks, and (3) clinical tasks. - An additional AYA social worker was recently hired to help offload some of LL’s clinical tasks. | - The hiring AYA-specific social worker and nurse practitioner represents an increase in staffing hours that might be allocated towards NA-SB. |
| **Oncologist** | - AS’s time is split between research (75%) and clinical (25%). Although this is a larger research focus than most of his colleagues have, most of them do have some time carved out for nonclinical tasks | - Oncology providers, particularly those doing research or other nonclinical tasks, may not have the time to deliver NA-SB. |
| **Organizational knowledge** | - Social workers have a robust knowledge of services and resources available at NCCH. | - Social workers may be particularly fit to facilitate the care coordination required by NA-SB. |
| **Knowledge and beliefs about the intervention** | - LL and AS are accustomed to assessing AYAs’ needs as part of routine care. | - LL and AS possess the fundamental knowledge and skills required to engage in a more formalized needs assessment process. |
| **Experience with PROMs** | - Providers had some experience administering PROMs. - Some AYAs have experience completing surveys. However, all AYAs were amenable to completing surveys as part of their cancer care. | - Providers’ experience administering PROMs will facilitate their ability to administer NA-SB. - Completing a PROM as part of their cancer care is acceptable to AYAs. |
| **Process** | | |
| **AYA Tasks** |  |  |
| Diagnosis | - After receiving a cancer diagnosis, AYAs are often in shock or in high distress, and may not know what their service needs are yet. | - NA-SB should not be administered immediately upon diagnosis. |
| Treatment schedules | - AYAs experience appointment fatigue during cancer treatment - AYAs struggle to balance their treatment schedules with competing priorities such as work, school, and raising children. | - NA-SB should be minimally burdensome to AYAs. - To the extent possible, services rendered should be embedded in existing treatment appointments, while patients are already in the hospital. - Note: in the context of COVID-19, NCCH’s AYA program has developed much more flexibility with virtual visits; this presents new opportunities to administer NA-SB virtually. |
| Outpatient appointments | - During outpatient visits, AYAs’ task load can be extensive. - Appointments entail some combinations of segments including transportation, parking, registration, labs, imaging, treatment, clinical | - NA-SB should accommodate the range of appointment types that AYAs have. - To the extent possible, NA-SB should incur no additional time burden to AYAs’ already long and exhausting appointments. |
| Inpatient stays | - Many AYAs have an inpatient stay at some point while they’re undergoing treatment - AYAs staying inpatient have lot of “deadtime” | - Capturing the needs of inpatient AYAs is important. - There is ample opportunity for NA-SB administration for AYAs staying inpatient. |
| End-of-life care | - For some AYAs, cancer is more than just a temporary hurdle. | - NA-SB content and delivery should be sensitive to those whose cancer is terminal. |
| **Caregivers’ tasks** | - AYAs rely heavily on their loved ones to navigate cancer care. | - NA-SB content should reflect the importance of changes that these relationships undergo after a cancer diagnosis. |
| **Provider tasks** |  |  |
| Identifying AYAs | - There is currently no systematic way for new AYA patients to be identified. | - As such, NA-SB should include a process or strategies for identifying new AYA patients (e.g., strengthening referral networks to expand reach). |
| Assessing AYAs’ needs | - For non-medical concerns, AYAs rely on the AYA social workers more than their oncology providers. Social workers are currently conducting needs assessments, although informally/conversationally | - NA-SB should leverage social workers’ existing workflow and expertise in the domain of needs assessment. |
| Documenting and communicating AYAs’ needs | - Providers use EPIC to document and communicate about patient information. - Providers also communicate via phone, email, pager and in-person. - AYAs are active users of MyChart. | - If possible, NA-SB should interface with EPIC in order to capitalize on existing systems. - NA-SB could also leverage existing communication channels (e.g., EMR messaging, pager, phone, etc.) - NA-SB could interface with MyChart. |
| Providing services and resources to meet AYAs’ needs | - AYAs report a number of barriers to service and resource use; some of these barriers (e.g., service capacity) may not be addressable by NA-SB. | - Flexibility should be built into NA-SB service provision to address downstream barriers to service use. For example, multiple services might be offered for a given need such that an AYA can select the option that is most feasible and appealing to them. |
| **Intervention** | | |
| **Goals for intervention** |  |  |
| Intervention outcomes | - Providers wanted NA-SB to fit seamlessly with existing workflows. They hoped it would formalize documentation and communication channels. - AYAs wanted the needs they report in NA-SB to be followed up on in a timely manner. | - To the extent possible, NA-SB should leverage and strengthen existing provider workflow, communication channels, and documentation practices. - Needs reported on NA-SB should trigger timely referral to appropriate services or other follow-up actions. |
| Needs assessment content | - AYAs reported many and diverse needs, but financial concerns were the most commonly discussed. | - In addition to capturing psychosocial concerns, peer support, transportation, and side effect management, it is particularly important that NA-SB capture financial needs which are pervasive among AYAs. |
| Timing | - Providers and AYAs wanted NA-SB to account for the dynamic nature of needs as AYAs move through treatment. Providers noted that AYAs’ needs persist into survivorship. - After receiving a cancer diagnosis, AYAs are often in shock or in high distress, and may not know what their service needs are yet. - AYAs appointments are long and exhausting. | - NA-SB should be administered at multiple timepoints throughout cancer including diagnosis, during treatment, at the end of treatment, and at some interval in survivorship. - NA-SB should not be administered immediately upon diagnosis when AYAs might not have a grasp of what they need yet. - In the clinic, NA-SB should be administered during times when AYAs’ wait times so as not to lengthen AYAs’ already long appointments. |
| Format | - AYAs did not express a strong preference in terms of paper versus electronic delivery of NA-SB. | - NA-SB could be administered through a paper or electronic format. |
| **Benefits of intervention** | - AYAs and providers expressed numerous potential benefits of NA-SB | - NA-SB potentially has high acceptability among prospective users. |
| **Costs of intervention** | - Providers expressed some potential costs of NA-SB including staffing time, workflow disruptions, and provider buy-in. - For AYAs, downstream barriers to service and resource exist (e.g., strict eligibility requirements, service capacity and timeliness) | - Staffing time, workflow disruptions, and provider buy-in represent potential barriers to implementation. - Additional strategies may be needed to address downstream barriers to AYAs’ use of services and resources. |

2.2 Interviews

**Table 9. Semi-structured interviews translation table**

| **Construct** | **Summary of contextual variation** | **NA-SB requirements** |
| --- | --- | --- |
| **Outer context** | | |
| **Patient characteristics** | - AYA programs may be housed in pediatric or adult oncology, and may serve varying age ranges. | The population reached by NA-SB may vary by institution. |
| **Cosmopolitanism** | - The structures and functions of AYA programs depend largely on their external network, or how they are positioned within their larger institutional network. | The referral networks established for NA-SB may depend on these external relationships. For example, AYA programs with satellite locations may need to consider whether AYAs receiving care in these satellite locations will be reached. |
| **Inner context** | | |
| **Structure** | - Institutions vary in terms of their model of AYA care. The development of AYA programs often occurs in a phased fashion. | In this context, the implementation of NA-SB may also occur in a phased fashion (e.g., initially delivered only to a certain disease or age group). |
| **Staffing** | - The availability of AYA-specific staff varies by institution. | Although NA-SB was developed to be usable and intuitive even to those without AYA expertise, programs without dedicated AYA staff may face additional challenges in implementing NA-SB. |
| **Funding** | - Some AYA programs rely initially on external foundation or grant funding; costs may eventually be absorbed by their health system if program staffing and activities prove beneficial. | Particularly for programs seeking internal funding for AYA programming, NA-SB could provide valuable patient-reported data to justify new or continued support of AYA programs. |
| **Physical space** | - The availability of designated spaces for AYAs varies by institution. | AYA programs with designated spaces for AYAs (e.g., AYA-specific infusion rooms) could use these spaces for NA-SB administration or service provision since they represent central locations where AYAs may be congregated. However, privacy is an important consideration. |
| **Functions** | - AYA programs are largely functioning as care coordination hubs for AYAs but face challenges to coordinating care for this population. | There is a need for tools like NA-SB to guide these care coordination efforts. |
| **Implementation climate** | - Buy-in from providers beyond just the AYA team is critical to the implementation of new interventions. | NA-SB implementation will require buy-in from providers beyond just the AYA team. Building this buy-in often requires some degree of culture change with respect to the perceived value-add of AYA-specific care. |
| **Leadership engagement** | - Leadership buy-in is also critical to the implementation of new interventions. | NA-SB implementation will require the articulation of a clear plan for implementation and a compelling case for the value of the intervention. |
| **Individuals** | | |
| **Related experience** | - Various provider types were responsible for assessing and following up on AYAs’ needs, but common characteristics of these individuals were reported. | The individual who delivers and follows up on NA-SB should have established relationships with providers to whom referrals will be made, assessment expertise, training in counseling, and rapport with AYAs. Although this may be social workers at NCCH, it varies among other institutions (e.g., child life specialists, nurse navigators). |
| **Attitudes towards NA-SB** | - Providers expressed largely positive attitudes towards NA-SB. | Providers may be receptive to NA-SB implementation. |
| **Process** | | |
| **Identifying AYA patients** | - Providers reported no systematic way for new AYA patients to be identified. | NA-SB should include a process or strategies for identifying new AYA patients (e.g., strengthening referral networks to expand reach). |
| **Assessing AYAs’ needs** | - Some providers’ programs were in the early phases of implementing a more formalized needs assessment process. | Success in these institutions suggests that NA-SB is feasible to implement. |
| **Documenting and communicating AYAs’ needs** | - Providers use the EHR to document and communicate about patient information. | If possible, NA-SB should interface with the EHR to capitalize on existing systems. However, working with software vendors to make the necessary changes represents a challenge to implementation. |
| **Providing services and resources** | - Some institutions do not have the capacity to respond to the full range of AYA needs. | Institutional service capacity represents a potential barrier to NA-SB implementation. A thorough understanding of existing services and resources should be gathered prior to implementation. |
| **Intervention** | | |
| **Goals for intervention** |  |  |
| Timing | - Providers noted that, given variation among AYAs and the unpredictability of clinical workflow, getting the needs assessment to AYAs at the opportune times would be really challenging. Furthermore, treatment trajectories vary tremendously, making it difficult to time re-assessment. They offered several suggestions about timing. | NA-SB should be administered at multiple timepoints throughout cancer treatment to capture the changing needs of AYAs, including diagnosis, during treatment, at the end of treatment, and at some interval in survivorship. It should not be administered immediately upon diagnosis when AYAs might not have a grasp of what they need yet. In the clinic, NA-SB should be administered during times when AYAs’ wait times so as not to lengthen AYAs’ already long appointments. |
| Format | - Providers did not express a strong preference about paper versus electronic delivery of NA-SB. | Although providers did not express a strong preference for NA-SB format, they recommended considering electronic delivery if NA-SB were to be delivered outside of the clinic. In the clinic, they noted several advantages of paper. |
| **Benefits of intervention** | - Providers expressed numerous potential benefits of NA-SB. | This suggests that NA-SB has high acceptability among prospective users. |

**3. Design team prototyping workshops**

3.1 Workshop #1

###### **Table 10. Design team decisions about CNQ-YP items up for elimination**

| **Item** | **Decision** | **Rationale** |
| --- | --- | --- |
| Being able to have a choice of times for appointments | Eliminate | Low importance (a choice of appointment times is typically “a given”) |
| Being able to have the same cancer treatment staff throughout treatment | Eliminate | Low actionability |
| Being able to have a choice of cancer care specialists | Eliminate | Low actionability |
| Knowing how much work I would miss | Eliminate | All work-related items into one item “*Navigating my work life while going through cancer treatment*” to avoid being unnecessarily specific. |
| Being able to have leisure spaces and activities | Eliminate | Low actionability in terms of leisure “spaces”. “Activities” covered by other items. |
| Knowing how to ask managers/coworkers for support | Revise | All work-related items collapsed into one item: “*Navigating my work life while going through cancer treatment*” to avoid being unnecessarily specific |
| Being able to get guidance about study options or future career paths | Revise | All school-related items collapsed into one item: “*Navigating my school life while going through cancer treatment*” to avoid being unnecessarily specific |
| Coping with my parent/s and/or partner being overprotective | Eliminate | Captured by other items: “*Coping with changes in my relationships with my family members*” and “*Coping with changes in my dating or romantic life*”.  Additionally, users said this was an issue to solved or navigated and not “coped” with. |
| Being able to be independent | Revise | Revised to “*Feeling independent*” to capture psychosocial implications. Physical independence captured by other items. |
| Having cancer treatment staff who treated me as an individual | Revise | Revised to “*Having cancer treatment staff who respected me as an individual, not just a cancer patient*” to capture a more specific and relevant concern. |
| Having cancer treatment staff who talked to me in private without my family | Revise | Revised to “*Having cancer treatment staff who offered to talk to me in private without my family*” to leave room for patient choice. |
| Having cancer treatment staff who could have a laugh with me | Eliminate | Low actionability |
| Being able to have pleasant surroundings at the cancer treatment center | Eliminate | Low actionability |
| Being able to have good food at the cancer treatment center | Eliminate | Low actionability |
| Being able to attend classes (while enrolled in school) | Eliminate | All school-related items collapsed into one item: “*Navigating my school life while going through cancer treatment*” to avoid being unnecessarily specific. |
| Being able to get extensions/special considerations (while enrolled in school) | Eliminate | All school-related items collapsed into one item: “*Navigating my school life while going through cancer treatment*” to avoid being unnecessarily specific. |
| Worrying about whether my treatment is working | Revise | Revised to “*Cancer treatment staff giving me honest and timely information about the status of my cancer treatment*” to more broadly capture transparent and timely communication during treatment.  Additionally, users noted that whether treatment is working is not binary. |
| Being able to have privacy at the cancer treatment center | Eliminate | Low actionability |
| Finding inner strength | Eliminate | Evokes the “fight against cancer” narrative (i.e., “toxic positivity”, which is not palatable to many AYAs |
| Having cancer treatment staff who were respectful | Eliminate | Low actionability; addressed by other items about cancer care team |
| Having cancer treatment staff who were approachable and friendly | Eliminate | Low actionability |
| Having cancer treatment staff who listened to my concerns | Revise | Revised to “*Having cancer treatment staff who asked about my concerns about treatment*” because AYAs often don’t broach their concerns with their providers (“you don’t know what you don’t know”) |
| Having cancer treatment staff who let me ask questions | Revise | Revised to “*Having cancer treatment staff who encouraged me to ask questions*” to make more patient-centered |
| Having cancer treatment staff who let me make decisions about my treatment | Revise | Revised to “*Having cancer treatment staff who engaged me in decision-making about treatment and respected my decisions*” to reflect the process, rather than the outcome.  Additionally, provider users noted that it’s not always possible to relinquish total control of decision-making. |
| Finding information that described relaxation techniques | Revise | Revised to “*Finding information on alternative therapies (herbal treatment, acupuncture, massage therapy, meditation, etc.)”* to make more inclusive of other modalities. |
| Being able to accept my diagnosis | Revise | Revised to “*Having what I need to cope with my diagnosis*” to make more actionable. |
| Cancer treatment staff telling me whether I would be able to have children | Revise | Broke this item into multiple items about fertility to be more comprehensive and less presumptuous regarding AYAs’ desire to have children:  *“Receiving information and counseling about risk for infertility and fertility preservation options”*  *“Receiving information on treating infertility and other options for having children (i.e., artificial insemination, in vitro fertilization, surrogacy, adoption, etc.)”*  *“Receiving information on the genetic inheritability of my cancer”* |

###### **Table 11. Design team decisions about potential additional items**

| **Item** | **Decision** | **Wording** |
| --- | --- | --- |
| Sexual health | Add | *Cancer treatment staff giving me information about sexuality and intimacy during cancer treatment* |
| Worrying about health insurance coverage | Add | *Worrying about my health insurance (e.g., access/eligibility, coverage, cost)* |
| Physical therapy | Don’t add | N/A |
| Being able to get guidance about financial aid or loan repayment | Add | *Getting guidance about scholarships or loan repayment options* |
| Other financial needs | Add | *Paying my bills*  *Having childcare during my cancer care appointments*  *Having stable housing* |
| Transportation | Add | *Getting to and from my cancer care appointments* |
| Coping with changes in my relationships with friends | Add | *Coping with changes in my relationships with friends* |
| If and how to tell my employer I have cancer | Don’t add | All work-related items collapsed into one item: *Navigating my work life while going through cancer treatment* |
| Sleeping | Add | *Sleeping well* |
| Spiritual needs | Add | *Having spiritual support or faith-based resources* |
| Alcohol and drug use | Add | *Cancer treatment staff giving me information about drug and alcohol use during cancer treatment*  *Having the resources I need to quit smoking* |

Figure 7. Concept mapping cluster map selected by design team


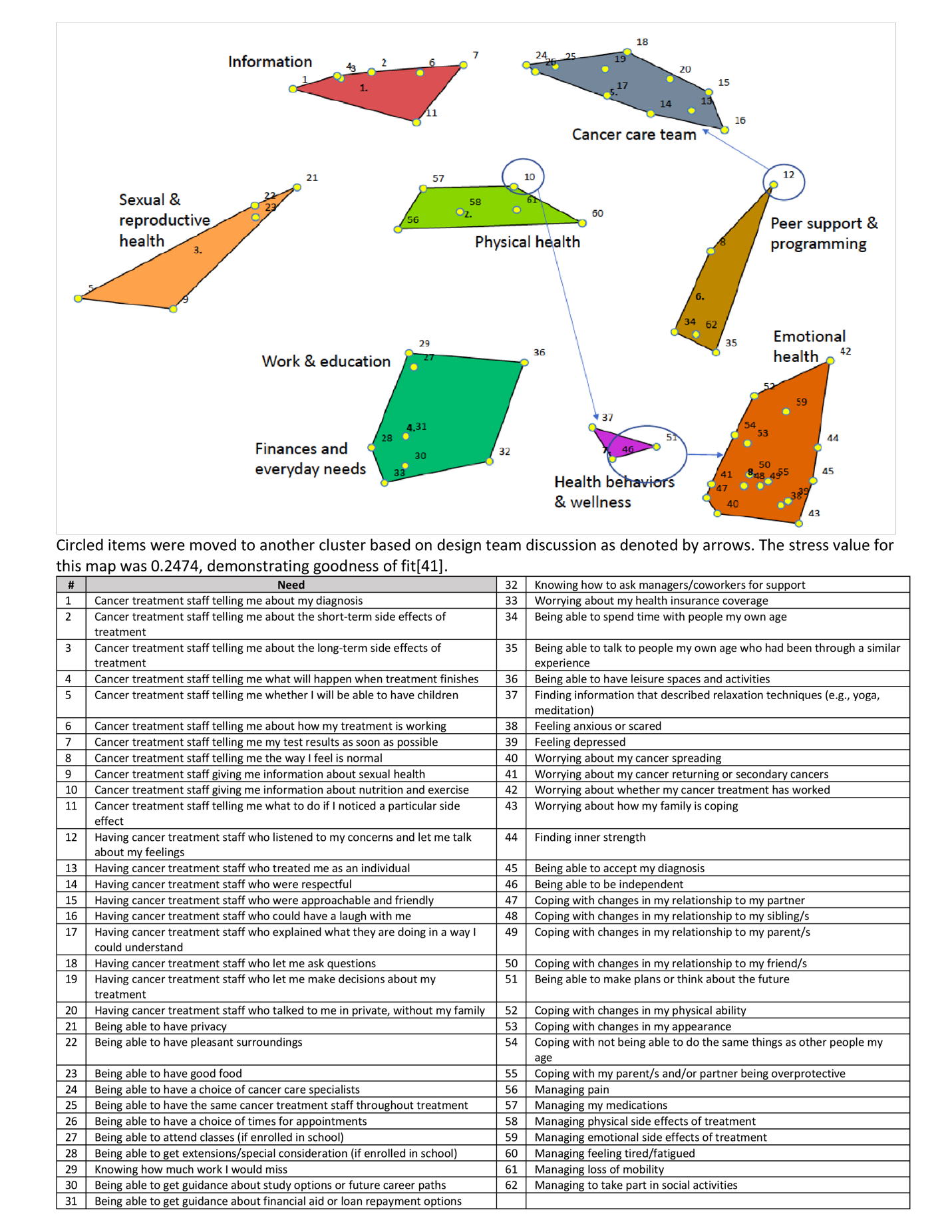


Table 12. Refined items by follow-up domain

| **Information** | | |
| --- | --- | --- |
| 1 | Cancer treatment staff telling me about my diagnosis | Cluster 1 |
| 2 | Cancer treatment staff telling me about the short-term side effects of treatment |  |
| 3 | Cancer treatment staff telling me about the long-term side effects of treatment |  |
| 4 | Cancer treatment staff telling me what will happen when treatment finishes |  |
| 5 | Cancer treatment staff giving me honest and timely information about the status of my cancer treatment |  |
| 6 | Cancer treatment staff telling me my test results as soon as possible |  |
| 7 | Cancer treatment staff telling me what to do if I noticed a particular side effect |  |
| **Cancer Care Team** | | |
| 8 | Having cancer treatment staff who respected me as an individual, not just a cancer patient | Cluster 5 |
| 9 | Having cancer treatment staff who offered to talk to me in private, without my family |  |
| 10 | Having cancer treatment staff who explained what they are doing in a way I could understand |  |
| 11 | Having cancer treatment staff who encouraged me to ask questions |  |
| 12 | Having cancer treatment staff who engaged me in decision-making about my treatment and respected my decisions |  |
| 13 | Having cancer treatment staff who asked about my concerns about treatment |  |
| **Physical Health** | | |
| 14 | Managing pain | Cluster 2 |
| 15 | Managing my medications |  |
| 16 | Managing physical side effects of treatment |  |
| 17 | Managing feeling tired/ fatigued |  |
| 18 | Managing loss of mobility |  |
| **Emotional Health** | | |
| 19 | Feeling anxious or scared | Cluster 8 |
| 20 | Feeling depressed |  |
| 21 | Having what I need to cope with my diagnosis |  |
| 22 | Worrying about my cancer spreading |  |
| 23 | Worrying about my cancer returning or secondary cancers |  |
| 24 | Worrying about how my family is coping |  |
| 25 | Coping with changes in my dating or romantic life |  |
| 26 | Coping with changes in my relationships with my family members |  |
| 27 | Coping with changes in my relationships with friends |  |
| 28 | Feeling independent |  |
| 29 | Coping with changes in my physical ability |  |
| 30 | Coping with changes in my appearance |  |
| 31 | Coping with not being able to do the same things as other people my age |  |
| 32 | Managing the emotional side effects of treatment |  |
| 33 | Being able to make plans or think about the future |  |
| **Sexual & Reproductive Health** | | |
| 34 | Receiving information and counseling about risk for infertility and fertility preservation options | Cluster 3 |
| 35 | Receiving information on treating infertility and other options for having children (i.e., artificial insemination, in vitro fertilization, surrogacy, adoption, etc.) |  |
| 36 | Receiving information on the genetic inheritability of my cancer |  |
| 37 | Cancer treatment staff giving me information about sexuality and intimacy during cancer treatment |  |
| **Health Behaviors & Wellness** | | |
| 38 | Cancer treatment staff giving me information about nutrition and exercise | Cluster 7 |
| 39 | Sleeping well |  |
| 40 | Having the resources I need to quit smoking |  |
| 41 | Cancer treatment staff giving me information about drug and alcohol use during cancer treatment |  |
| 42 | Having spiritual support or faith-based resources |  |
| 43 | Finding information on alternative therapies (herbal treatment, acupuncture, massage therapy, meditation, etc.) |  |
| **Work & Education** | | |
| 44 | Navigating my school life while going through cancer treatment | Cluster 4 |
| 45 | Navigating my work life while going through cancer treatment |  |
| **Peer Support & Programming** | | |
| 46 | Being able to spend time with people my own age | Cluster 6 |
| 47 | Being able to talk to people my own age who have been through a similar experience |  |
| 48 | Managing to take part in social activities |  |
| **Finances & Everyday needs** | | |
| 49 | Paying my bills | Cluster 4 |
| 50 | Getting guidance about scholarships or loan repayment options |  |
| 51 | Worrying about my health insurance (e.g., access/eligibility, coverage, cost) |  |
| 52 | Getting to and from my cancer care appointments |  |
| 53 | Having childcare during my cancer care appointments |  |
| 54 | Having stable housing |  |

3.2 Workshop #2

**Figure 8. Design team member rankings of potential barriers to NA-SB implementation**

**
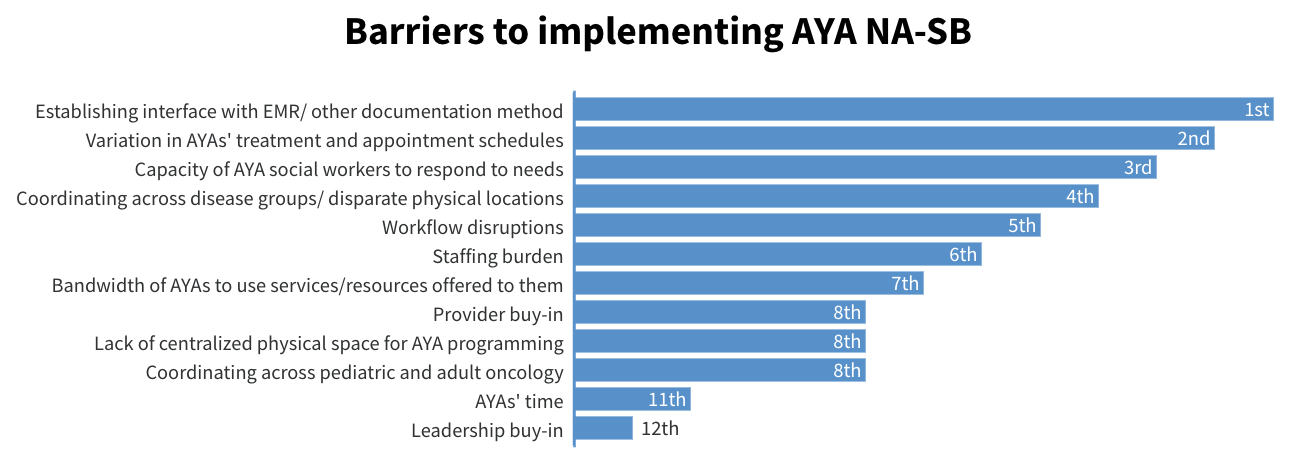
**

**Table 13. Implementation strategies identified by design team**

| **Implementation barrier** | **Implementation strategies** |
| --- | --- |
| **Outer context** | |
| Administration challenges resulting from variation in AYAs’ treatment and appointment schedules | During pilot testing, include three different arms with different intervals of administration: (1) every month, (2) every 3 months, and (3) with each significant change in clinical status. |
| AYAs’ limited time | Identify approach for ensuring that services and resources are provided in a timely manner once needs are identified so that AYAs promptly incur intervention benefits. |
| Limited bandwidth of AYAs to use services and resources offered to them | Flexibility should be built into NA-SB service provision to address downstream barriers to service use. For example, multiple services might be offered for a given need such that an AYA can select the option that is most feasible and appealing to them.  To the extent possible, services rendered might be embedded in existing treatment appointments, while patients are already in the hospital. |
| **Inner context** | |
| Lack of interface with EMR/ other documentation method | Use pilot testing to establish a blueprint for EMR modifications needed  Where possible, leverage existing communication and documentation channels in NA-SB delivery. Modify communication and documentation processes as needed to allow for, at a minimum, traceable documentation of follow-up on needs. |
| Limited institutional capacity to respond to needs | Obtain a thorough understanding of services and resources available at your institution prior to implementation, identifying gaps that may hinder follow-up on needs reported by AYAs. For identified gaps, bolster existing services or tailor the needs assessment to address the subset of needs that your institution has the capacity to address.  Use pilot testing to tailor NA-SB to your institution, for example, to make additional refinements to the needs assessment tool to tailor it to your institution.  Set clear expectations about follow-up when NA-SB is first introduced to AYAs. |
| Challenges coordinating across disease groups/disparate physical locations | Consider a phased-in approach to implementation, for example, by implementing within one disease group and expanding outwards.  Provide education across disease groups and identify champions within each disease group to facilitate referrals of AYAs to AYA program. |
| Workflow disruptions | Explicitly outline referral pathways for each follow-up domain, identifying primary contacts, current workflow, and best method of communication for service/resource providers. |
| Staffing burden | Leverage staff who are currently assessing and addressing the needs of AYA patients at your institution and thus, have the necessary expertise and time allocated towards these tasks. |
| Lack of centralized space for AYA programming | NA-SB delivery should be flexible in terms of location to accommodate disparate physical locations. |
| Challenges coordinating across adult and pediatric oncology | Provide education across clinics and identify champions within each to facilitate referrals of AYAs to AYA program. |
| Low leadership buy-in | Build buy-in among leadership, emphasizing the potential benefits of NA-SB for patient care, patient-provider communication, provider-provider communication, and program development. |
| **Individual characteristics** | |
| Low provider buy-in | Build buy-in by engaging in implementation planning any provider groups who will interface with NA-SB in practice. |
